# Programmable MRI contrast switching for spatiotemporal mapping of thrombus maturation via enzyme-directed nanoprobe reconfiguration

**DOI:** 10.1186/s40580-025-00518-w

**Published:** 2025-10-30

**Authors:** Chi Lin, Fang-Yu Hsu, Chun-Ming Shih, Tsai-Mu Cheng, Alexander T. H. Wu, Chia-Hsiung Cheng, Hsin-Ying Lu, Chun-Che Shih, Fwu-Long Mi

**Affiliations:** 1 Department of Biochemistry and Molecular Cell Biology, School of Medicine, College of Medicine, Taipei Medical University, Taipei, 11031 Taiwan; 2 Graduate Institute of Medical Sciences, College of Medicine, Taipei Medical University, Taipei, 11031 Taiwan; 3 Taipei Heart Institute, Taipei Medical University, Taipei, 11031 Taiwan; 4 Division of Cardiology and Cardiovascular Research Center, Taipei Medical University Hospital, Taipei, 11031 Taiwan; 5 The PhD Program for Translational Medicine, College of Medical Science and Technology, Taipei Medical University, Taipei, 11031 Taiwan; 6 Division of Cardiovascular Surgery, Department of Surgery, Wan Fang Hospital, Taipei Medical University, Taipei, 11031 Taiwan; 7 Department of Surgery, School of Medicine, College of Medicine, Taipei Medical University, Taipei, 11031 Taiwan; 8 Graduate Institute of Nanomedicine and Medical Engineering, College of Biomedical Engineering, Taipei Medical University, Taipei, 11031 Taiwan

**Keywords:** Programmable MRI nanoprobe, MMP-responsive contrast switching, Gelatin-based structural modulation, Thrombus maturation mapping, Targeted theranostic delivery, Fucoidan

## Abstract

Spatiotemporal mapping of thrombus remodeling remains a major unmet challenge due to the lack of diagnostic tools capable of dynamically converting local biochemical activity into quantitative imaging signals. Current clinical methods lack sensitivity and specificity for accurate thrombus staging. Here, we present a programmable MRI nanoplatform enabling enzyme-gated dual-mode contrast switching for dynamic thrombus profiling and guided thrombolysis. The nanoprobe achieves broad-spectrum thrombus targeting by recognizing two complementary biomarkers uniquely expressed at distinct thrombus maturation stages, and integrates gelatin-guided structural reconfiguration with magnetic nanoparticle clustering to modulate MRI contrast. Gelatin modulates the nanoprobe structure, restricting water proton accessibility and promoting internal densification, thereby synchronously suppressing T1-weighted signals and amplifying T2-weighted contrast. Upon activation by thrombus-associated MMP-2/9, the nanoprobe disassembles, reversing its nano-architecture and signal behavior. This smart signal transformation quantitatively correlates with MMP activity, thrombus age, and collagen content, generating stage-dependent T1/T2 ratios. The nanoprobe also enables enzyme-triggered fibrinolytic release, achieving site-specific thrombolysis with minimal hemorrhagic risk. This materials-based strategy translates dynamic microenvironmental remodeling into high-resolution MRI outputs, establishing a programmable framework for precision imaging and therapy. These programmable imaging outputs support data-driven diagnostics, enable clinical treatment stratification, and offer a standardized reference for modeling enzyme-rich pathological environments.

**Graphical Abstract:**

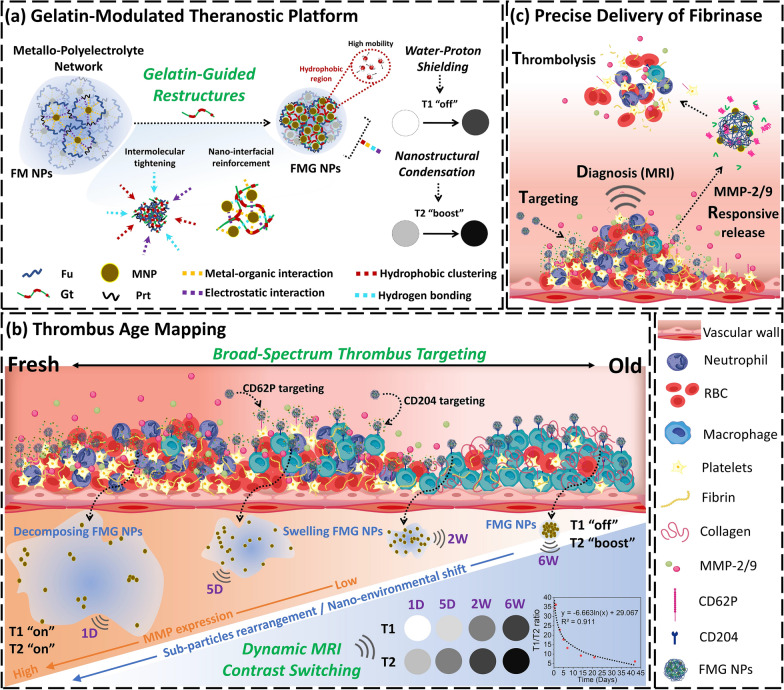

**Supplementary Information:**

The online version contains supplementary material available at 10.1186/s40580-025-00518-w.

## Introduction

Translating pathological microenvironment signals into imaging-readable outputs remains a critical challenge in responsive materials design, especially for dynamic disease states such as thrombosis. Thromboses are a primary contributor to many life-threatening cardiovascular diseases which constitute leading factors in global levels of morbidity and mortality. The composition of a thrombus evolves over time due to complex interactions among coagulation factors, leukocytes, cytokines, proteases, and other factors. Various classification criteria exist based on the different stages of a thrombosis. Generally, based on temporal compositional changes, thrombi can be roughly categorized into different stages, including acute/early/fresh (< 1 days), lytic (1–5 days), infiltrating/organized (4–7 days), medium/organized (1–3 weeks), and old/organized (> 6 weeks) [1–3]. Compositional differences among these stages are substantial, and represent distinct pathological tissue entities.

The ages or tissue statuses of thrombi significantly influence the progression of thrombus-related diseases, the selection of treatment strategies, and treatment outcomes [4]. Fibrinolytic therapies exhibit significantly better thrombolysis efficacies in fresh thrombi compared to aged thrombi [5]. Additionally, fibrinolytic therapy has not only demonstrated limited efficacy in aged thrombi but also exposes patients to life-threatening bleeding risks [6]. Mechanical thrombectomy treatment is also affected by the different compositions of thrombi, which impact risk assessments and treatment efficacies. Thrombi with a lower fibrin density or less organization are more easily recanalized [7, 8]. However, they are also more prone to fragmentation during an intervention, which can lead to more fatal myocardial infarctions, ischemic strokes, and pulmonary embolisms [6]. Even after successful thrombus clearance with a mechanical thrombectomy, older thrombi contribute to prolonged reperfusion times [9, 10]. Furthermore, clinical reports have demonstrated that approximately two-thirds of coronary artery thrombi in acute coronary deaths exhibit organization [3]. These findings demonstrate that the varying maturities of thrombi are a crucial factor determining treatment outcomes. Thus, having precise information about the age of a thrombus is essential for determining appropriate treatment plans and methods, preventing potential complications, and developing tailored therapeutic strategies for thrombi at different stages.

Currently, a patient’s clinical history serves as the primary method for assessing a thrombus’ age. However, many clots remain asymptomatic or undetectable within days or weeks of thrombosis, rendering patient histories an inconsistent tool for evaluating thrombus ages [11]. Non-invasive imaging techniques have been attempted for thrombus diagnosis, with potential applications in clinical settings for distinguishing thrombus ages. These techniques include ultrasound elastography (US) [12, 13] and specialized magnetic resonance (MR) imaging techniques [1, 14]. Unlike US, which requires good acoustic windows, struggles with deeper or heterogeneous tissues, relies on a reference point [15], and is operator-dependent due to the need for external compression [16], MRI offers several advantages. MRI provides excellent tissue penetration depth, high spatial resolution, non-ionizing radiation, non-invasiveness, and the ability to track treatment progress [17, 18]. Specialized MRI techniques have been developed, including MR direct thrombus imaging (MRDTI) or black-blood MRI sequences, for discriminating thrombi of different ages [1, 14]. However, both techniques require specific software and hardware support and have not yet been widely adopted [15]. Furthermore, there is room for improvement in their discriminatory and recognition capabilities, especially since diagnosing smaller-vessel thrombus using MRI alone remains challenging [17].

To improve imaging contrast and clarity, refining MRI contrast agents through lesion-specific targeting or responsiveness is a popular research focus [17]. Currently, MRI contrast is enhanced by targeting activated platelets through specific binding with glycoprotein IIb (GPIIb)/IIIa [19, 20], integrin αIIbβ3 [21], or annexin V [22], as well as by targeting fibrin [23]. However, these approaches can only enhance signals in early thrombi and cannot effectively distinguish between non-thrombotic and aged thrombi, let alone diagnose the age of a thrombus. For lesion-specific responsiveness, certain enzymes, including thrombin, MMPs, and tissue inhibitors of metalloproteinase (TIMPs), have been identified as biomarkers for determining thrombus ages, with MMP-2 and MMP-9 showing a gradual decrease in expression levels with age [2, 24]. Unfortunately, research demonstrating sufficient evidence for the development and application of non-invasive diagnostic agents incorporating these biomarkers is still lacking. These changes in the thrombus microenvironment inspired us to believe that leveraging these differences could lead to the development of a novel nanomaterial capable of reflecting age information in response to MMP-2 and MMP-9. To achieve the goal of using MRI contrast agents to diagnose the age of thrombi, challenges had to be overcome, including developing methods to target thrombi of all ages and establishing a platform to translate these markers into MR images.

Previous studies, including our own and others, utilized activated platelets for targeted delivery therapy to thrombi, achieving efficient targeting efficiency by targeting the high expression of p-selectin (CD62P) on their surface [25–27]. On the other hand, organized aged thrombi exhibit positive expression of M2 macrophage markers, such as CD163, scavenger receptor A (CD204), and CD206, with CD204-positive cells beginning to infiltrate from the mixed stage and continuing to abundantly infiltrate into the fibrin-rich and organizing stages [28, 29]. This suggested that the development of a dual-targeting thrombus delivery system, targeting CD62P in the early to middle stages and CD204 in the middle to late stages, holds promise for achieving targeted delivery across all ages of thrombi. Fucoidan (Fu), a sulfated polysaccharide derived from brown algae, was demonstrated in our and others research to possess an excellent thrombus/CD62P-targeting capability and is widely used in thrombus drug delivery [25–27]. Fu also exhibits notable affinity for CD204, which is present on the surface of macrophages [30, 31]. Mechanistically, its sulfated fucose residues mimic natural selectin ligands, enabling specific binding to CD62P on activated platelets [25–27]. Meanwhile, CD204 scavenger receptors recognize its sulfated polyanionic structure, whose spatial and electrostatic features favor pattern recognition, as demonstrated by competitive binding assays in macrophages [30]. These dual interactions facilitate the retention of Fu-based nanoparticles at thrombus sites and enhance site-specific drug delivery across different stages of thrombus maturation. Therefore, utilizing Fu as a targeting molecule, which targets both early thrombi containing activated platelets expressing CD62P and old thrombi containing macrophages expressing CD204, has the potential to meet the demand for targeting thrombi of all ages.

Utilizing activatable MRI contrast agents, such as degradable polymer matrices encapsulating Gd oxide nanoparticles or superparamagnetic iron oxide nanoparticles (MNPs), to modulate changes in the aqueous environment and neighbor distance to amplify or attenuate MR contrast, known as magnetic relaxation switchability, has emerged as a promising tool [32–35]. Dual-switchable MRI contrast agents are more powerful and are designed to simultaneously alter T1- and T2-weighted signals through specific biological events. This configuration allows the agents to reflect differences in the lesion environment and provide signal enhancement for MRI contrast in all states, making them more suitable for simultaneously diagnosing the location and age of thrombi.

To address the need for responsive and structurally adaptive MRI nanoprobes, we employed MNPs and gelatin (Gt) to develop a dual-switchable MRI contrast nanocomplex. Gt, a collagen-derived biopolymer known for its excellent biocompatibility, exhibits intrinsic amphiphilic properties and abundant chemically active functional groups derived from its amino acid residues [36, 37]. These characteristics enable Gt to facilitate hydrophobic domain formation, intermolecular interactions, and protease-sensitive structural modulation, making it particularly well-suited for engineering MRI-responsive nanostructures. Despite advances in molecular imaging, current MRI nanoprobes rarely couple endogenous enzymatic activity with signal transformation for thrombus staging. Here, we harness Gt-mediated architectural control and MMP-responsiveness to develop a programmable nanoprobe that synchronizes nanostructural remodeling with dual-mode MRI output for precise thrombus profiling. While its enzymatic degradability by MMP-2 and MMP-9 is well established, the potential of Gt to modulate nanoprobe architecture and dynamically influence MRI contras, through multi-interaction-driven nanoassembly and enzyme-triggered remodeling, remains largely unexplored. Neighbor distances among MNPs can regulate the saturation magnetization and magnetic resonance T2 relaxation of aggregates, ultimately leading to improved MRI contrast effects, which increase with a decrease in the neighboring distance [35]. In T1-weighted images, distances among MNPs have a relatively minor impact on the T1 contrast [38]. However, the hydrophobic layer can serve as an outer barrier, hindering interactions with water molecules, thereby reducing T1-weighted imaging contrast [32, 33]. Despite the lack of utilizing nanoparticles responsive to MMP-2 or MMP-9 in thrombus treatment or diagnosis to date, research endeavors have led to the development of nanoparticles that have shown promise in non-invasive imaging, as exemplified by hindlimb ischemic fluorogenic nanoprobes [39] and MR-tuned probes tailored to detect tumors and bacterial infections [33, 40]. Among these, nanoparticles composed of Gt were designed to disintegrate in environments with high MMP-2/MMP-9 expressions, thereby deforming and releasing their payload [41, 42]. This deformation alters neighbor distance among MNPs to achieve MMP-2/MMP-9-triggered distance-dependent MR tuning, thereby establishing the corresponding T1/T2 ratio for each thrombus age stage.

In this study, we present a programmable theranostic nanoplatform that integrates MMP-responsive dual-mode MRI contrast switching with broad-spectrum thrombus targeting (Scheme 1a). By leveraging the stage-specific expression patterns of CD62P and CD204, Fu was employed as a targeting ligand to achieve comprehensive thrombus recognition across early to organized stages (Scheme 1b). Concurrently, Gt and MNP were engineered into a dynamic assembly capable of enzyme-triggered structural reconfiguration, translating thrombus-associated MMP activity into quantifiable T1/T2 signal modulation. Furthermore, the platform was extended to encapsulate urokinase (UK), a clinically approved plasminogen activator with a well-defined mechanism and broad clinical use, as a representative fibrinolytic agent to evaluate the nanocarrier’s delivery performance, enabling MMP-triggered drug release precisely at thrombus sites [43–46]. This approach addresses key limitations of conventional thrombolytic therapy, such as rapid clearance and systemic bleeding risk, by enhancing site-specific thrombolysis efficiency and safety [43–46] (Scheme 1c). Together, this multifunctional strategy enables simultaneous thrombus staging, localization, and treatment, offering a unified framework for precision thrombus management. This design-centric approach bridges biochemical responsiveness with imaging-guided personalized therapy, laying the foundation for next-generation cardiovascular theranostics.

**Fig. 1:**
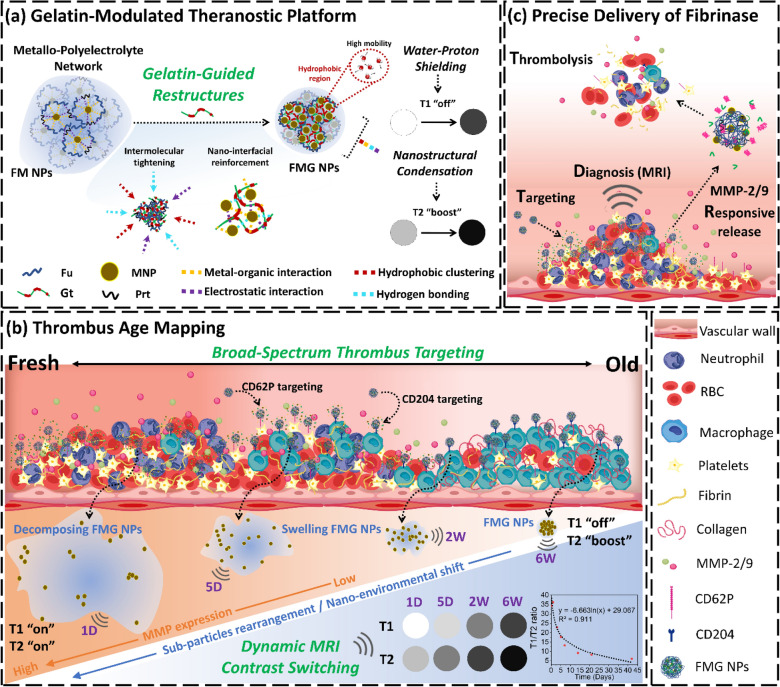
Fabrication of a gelatin-modulated theranostic platform for precision thrombus management. **a** Leveraging the unique functional groups of Gt to mediate coordinated interactions, including intermolecular tightening and nano-interfacial reinforcement, resulting in water proton shielding and MNP compaction. These structural modifications modulate MRI contrast via water proton shielding and magnetic dipole interactions, while also facilitating MMP responsiveness by enabling enzyme-triggered disassembly and contrast switching, as reflected in T1/T2 signal modulation. **b** MMP-driven conversion of thrombus age information into MRI signals, achieved through a multistage thrombus-targeting strategy and nanostructure-mediated dynamic MRI contrast switching. **c** A thrombus-targeting, MRI-diagnosis, and MMP-sensitive nanodelivery system for precise Uk release, enhancing thrombolytic efficiency while minimizing hemorrhagic risks

## Results and discussion

All experimental methods and characterization details are provided in the Supporting Information.

### Gelatin-mediated nanoarchitecture modulation for MRI contrast enhancement

A Gt-modulated nanoformulation was developed to achieve dynamic MRI contrast switching and thrombus-targeted delivery. By leveraging the structural versatility of Gt, this formulation modulates nanoprobe architecture to enhance MMP-responsive structural adaptation and optimize MRI contrast properties. Gt served as a key structural modulator, imparting additional intermolecular interactions that enhanced nanoprobe compaction and sub-particle packing, thereby optimizing MRI contrast properties. The fabrication process was refined to achieve a stable nanoprobe with well-defined physicochemical characteristics, ensuring a balance between nanoparticle size, surface charge, and MMP responsiveness.

**Fig. 2:**
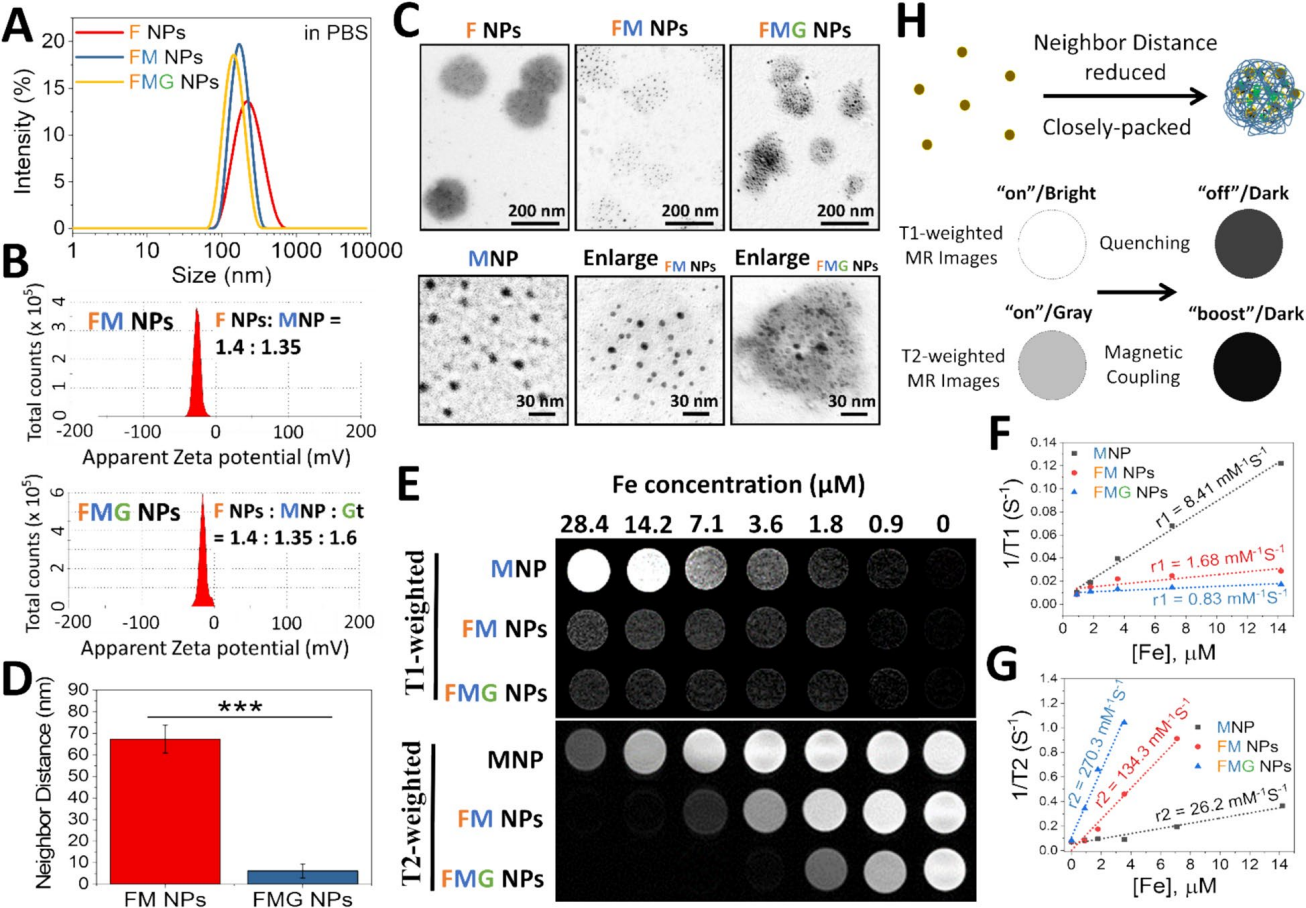
Fabrication and characterization of FMG NPs. **A** Particle size distribution. **B** ζ-potential distribution. **C** TEM images. **D** Neighbor distances analyzed from TEM images using ImageJ and R software (*n* = 20). **E** T1- and T2-weighted MR images. **F**, **G** Corresponding quantitative analyses (*n* = 3). **H** Schematic of water proton shielding and magnetic dipole coupling mechanisms driving dual-mode MRI contrast transformation. Data are expressed as mean ± SD. Statistical significance was assessed using Student’s t-test (n.s., non-significant difference, *** *p* < 0.001)

To construct this nanoprobe, we first assembled a polyelectrolyte-based nanoformulation (F NPs) through the complexation of Fu and protamine (Prt), which provided a stable yet adaptable nanostructure. Magnetic nanoparticles (MNPs), which have an approximate particle size of 10 nm, a highly negative surface charge of − 38.3 mV, and a saturation magnetization of 58.4 emu/g (Figure S1A-C), were then assembled into this framework in an optimal balance, forming FM NPs, which exhibited a well-defined morphology with a monomodal size distribution (Fig. 2A), an average particle size of 170.2 nm, a polydispersity index (PDI) of 0.11, and a surface charge of − 25.6 mV (Fig. 2B, Figure S2, Table S1). Transmission electron microscopy (TEM) revealed a transition from uniform spherical particles to a denser, sub-nano-clustered architecture (Fig. 2C, 2), confirming the effective integration of MNPs. This structural adaptation significantly influenced MRI contrast properties, where at an Fe-equivalent concentration of 14.2 µM, T1-weighted MRI signals decreased by 77.3% (r_1_ = 1.68 mM^−1^ s^−1^), while T2-weighted signals increased by 288% (r_2_ = 134.3 mM^−1^ s^−1^) (Fig. 2E–G). This phenomenon was attributed to reduced interaction between the MRI contrast agents and water molecules due to hindrance by an outer barrier, which limited hydrogen bonding, chelation, or diffusion with surrounding water molecules, thus suppressing T1-weighted signal enhancement [32, 33]. For T2-weighted signals, particularly with MNP-based agents, it is challenging to “quench” the strong superparamagnetic nature or magnetic moment of the nanoparticles [33, 34]. In contrast, the significantly reduced interparticle distances among MNPs enhanced magnetic dipole interactions, leading to a dramatic increase in T2-weighted MRI signals [35] (Fig. 2H).

**Fig. 3:**
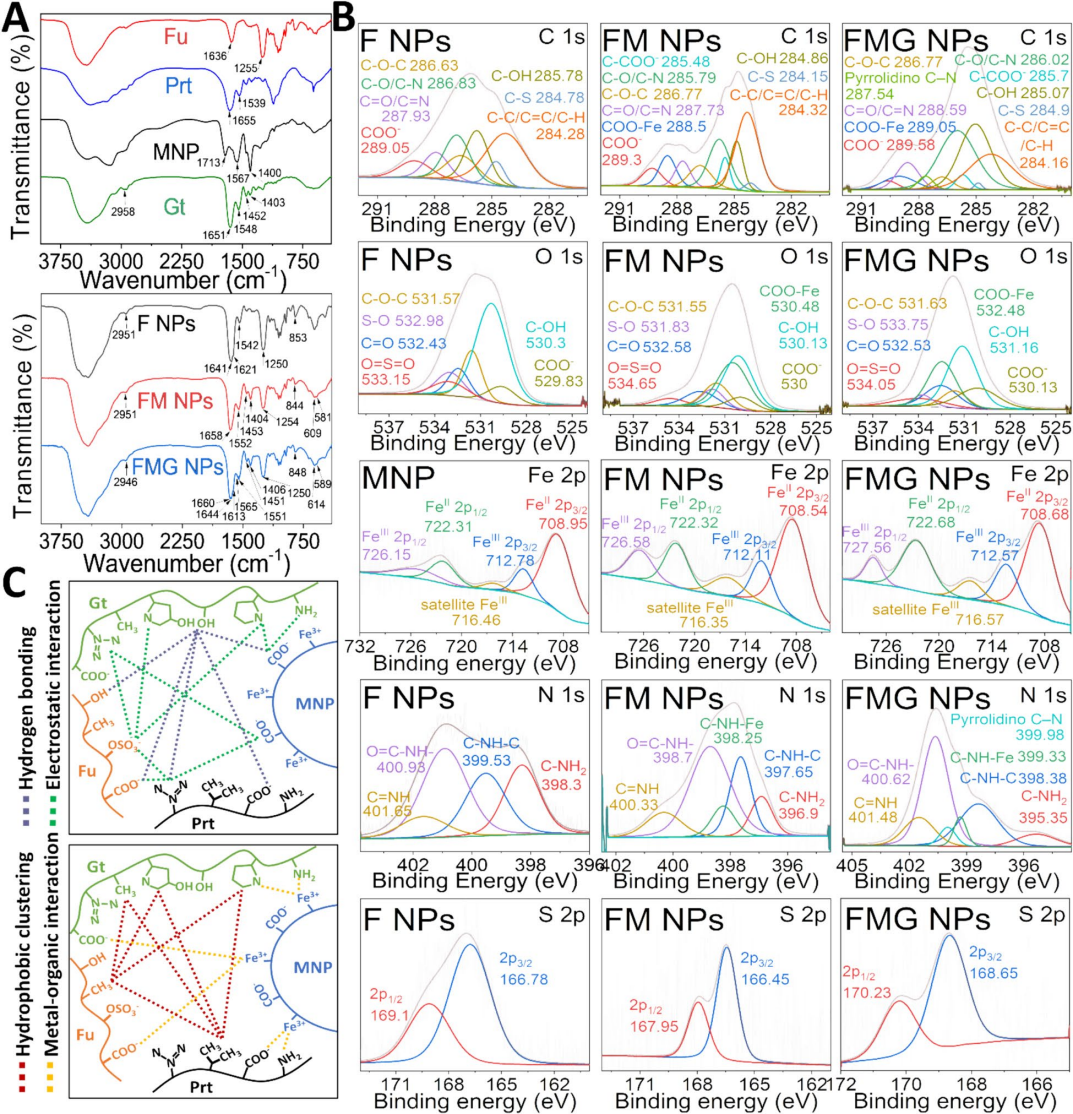
Characterization of the nanoparticles. **A** FT-IR spectrum. **B** XPS analysis including survey spectrum and high-resolution C1s, O1s, N1s, Fe 2p, and S2p spectra. **C** Illustration of the major molecular interactions involved in the assembly of FMG NPs

**Fig. 4:**
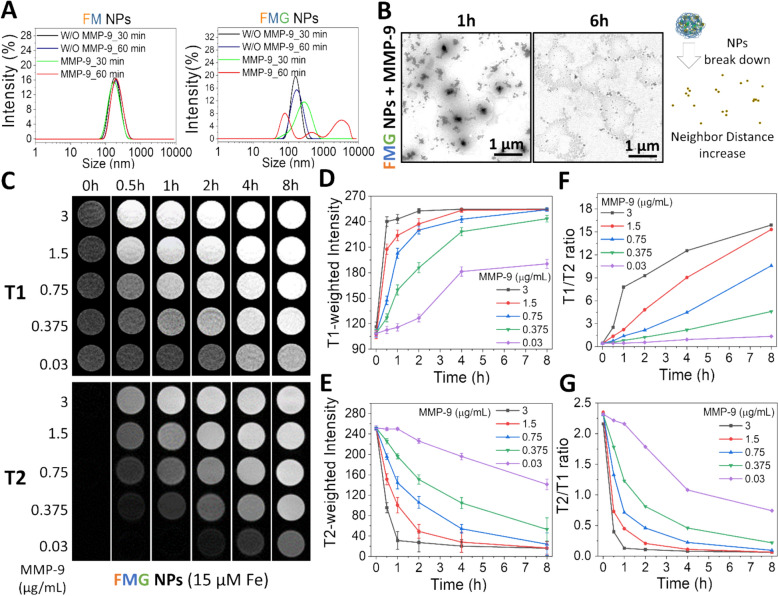
MMP sensitivity of FMG NPs. **A** Size distribution of FM NPs and FMG NPs in pH 6.8 PBS at 37 °C, with or without MMP-9 (0.75 µg/mL). **B** TEM images of FMF NPs with MMP-9 (0.75 µg/mL). Schematic depicting gelatin-induced structural compaction that simultaneously enhances T2 relaxivity via magnetic coupling and suppresses T1 signals through water proton shielding. **C** T1-weighted and T2-weighted MR images of FMG NPs (15 μM Fe equivalent) with MMP-9 (0.03–3 μg/mL) in pH 6.8 PBS at 37 °C. **D–G** Corresponding quantitative analyses and calculations (*n* = 3). Data are expressed as mean ± SD

Building on this FM NP platform, Gt was introduced as a key structural regulator, transforming the nanoprobe into a dynamic system capable of modulating its physicochemical and imaging properties. The incorporation of Gt induced substantial structural changes, reducing particle size from 170.2 nm to 144.5 nm as Gt content increased (0%–42% wt/wt, Gt/NPs) (Figure S3, Table S1). This reduction is likely attributable to structural compaction induced by Gt. As an amphiphilic protein bearing positively charged residues under physiological conditions, Gt enhances electrostatic interactions with the anionic Fu backbone and may also provide hydrophobic associations within the nanocomplex [36, 37]. These interactions are expected to result in denser molecular packing, reduced hydration, and a reduced tendency toward aggregation during self-assembly, ultimately yielding smaller and more uniform nanoparticles. In FM NPs, this compaction may be further reinforced by coordination between Gt residues and MNP surfaces, as supported by XPS analysis (see also Fig. 3B). Simultaneously, the surface potential significantly decreased (Figure S3C), which was attributed to the numerous positively charged groups, such as guanidinium and amine groups, present on Gt. To balance sufficient surface Fu for targeting capability with adequate Gt for a rapid response in MMP-2/9 environments, the minimum Gt ratio that optimizing MMP-2/9 sensitivity was identified. Testing revealed that a Gt content of 36.8% (wt/wt, Gt/NPs) yielded the strongest response to MMP-9. When particles contained 30.4% Gt, their size distribution widened over time but consistently remained unimodal, indicating a swollen state instead of disintegration (Figure S3D). With 36.8% Gt, FMG NPs exhibited a broad, asymmetric peak within 30 min of MMP-9 treatment, progressing to a multimodal distribution after 60 min (Fig. 4A). Consequently, the nanoparticle formulation with a weight ratio of F NPs: MNP: Gt of 1.4: 1.35: 1.6, designated FMG NPs, was selected. These FMG NPs demonstrated an average particle size of 152.4 nm with a PDI of approximately 0.13 (Table S1), a surface charge of about −16.8 mV, a monomodal size distribution (Fig. 2A), and stability that was maintained under physiological conditions (Figure S3E). Incorporating Gt not only reduced the particle size but also enhanced the MNP encapsulation rate, which increased from 66.8% to 82.3%, with an encapsulation ratio of 0.27 mg MNP per mg FMG NPs (Table S1). In TEM images, where the MNP density was depicted as black-gray spheres, the matrix of FM NPs appeared grayish-white, while FMG NPs were dark gray (Fig. 2C). Additionally, the interparticle distance among packed MNPs significantly decreased, by approximately 10.9-fold (Fig. 2D), indicating enhanced magnetic dipole interactions at the nanoscale. These nanostructural rearrangements markedly amplified T2-weighted MRI signals. At an Fe-equivalent concentration of 3.6 μM, FMG NPs exhibited an r_2_ value of 270.3 mM^−1^s^−1^, corresponding to a 10.1-fold and 2.3-fold increase compared to MNPs and FM NPs, respectively (Fig. 2G). In contrast, T1-weighted MRI signals were strongly suppressed in FMG NPs, with an r_1_ value of 0.83 mM^−1^s^−1^, representing a 10.1-fold and 2.0-fold reduction relative to MNPs and FM NPs, respectively (Fig. 2F). This pronounced T1 signal attenuation may be attributed to the formation of hydrophobic internal regions induced by Gt [36, 37], which restricts water proton accessibility to the magnetic core, a mechanism commonly referred to as water proton shielding.

As shown in Fig. 3A, FTIR analysis revealed that the characteristic sulfate peaks of Fu at approximately 1250 and 850 cm^−1^ were present in F NPs and exhibited slight shifts upon incorporation of MNPs and Gt, indicating the involvement of sulfate ester groups in charge-driven self-assembly. In the spectrum of MNPs, a peak at 1713 cm^−1^ was attributed to C=O stretching of uncoordinated carboxylic acid groups. This peak disappeared in FM NPs, while the intensity near 1552 cm^−1^ increased, suggesting the formation of COO^−^-Fe coordination. Moreover, A peak near 1621 cm^−1^, tentatively assigned to the C=N stretching vibration of guanidinium groups, was observed in F NPs but disappeared upon MNP incorporation, suggesting that Prt participated in electrostatic interactions with anionic groups such as COO^−^ or sulfate esters. Upon Gt incorporation (FMG NPs), the amide I and II regions exhibited peak splitting and red shifts, with new amide I bands appearing at 1660 and 1644 cm^−1^ and amide II bands at 1565 and 1551 cm^−1^, reflecting rearrangement of Gt secondary structures and altered hydrogen bonding networks. The CH_3_ stretching vibration also shifted from 2958 cm^−1^ in Gt to 2946 cm^−1^ in FMG NPs, suggesting hydrophobic interactions between Gt side chains and other components such as Fu or Prt. Additionally, the appearance of Fe–O and Fe–N stretching vibrations at 614 and 589 cm^−1^, respectively, further confirmed the coordination of Gt with Fe ions on the MNP surface. Collectively, these spectral changes indicate that the formation of FMG NPs is driven by multivalent interactions including sulfate-guanidinium electrostatics, COO^−^-Fe coordination, hydrogen bonding, and hydrophobic associations. These interactions were further supported by XPS analysis (Fig. 3B).

Figure 3B presents XPS spectra of the nanoparticles and raw materials, providing valuable insights into molecular interactions that were driving structural changes within the nanoparticles. These structural changes notably altered neighbor distances of MNPs, resulting in significant inversion of MRI T1/T2 signals. The comprehensive XPS spectra of F NPs, FM NPs, and FMG NPs confirmed the presence of C, N, O, Fe, and S elements, indicating the successful integration of their components. The nanoparticles were primarily assembled through electrostatic interactions, coordination reactions, and hydrogen bonding.

The deconvoluted C 1 s spectrum of F NPs revealed characteristic peaks at 284.2 (C–C/C=C/C–H), 284.7 (C–S), 285.7 (C–OH), 286.6 (C–O–C), 286.8 (C–O/C–N), 287.9 (C=O/C=N), and 289 eV (COO⁻), corresponding to the sulfate ester and uronic acid units of Fu, as well as the carbon backbone and guanidine group of Prt. Similarly, the MNP spectrum showed deconvoluted peaks at 284.9 (C–C/C–H), 285.6 (C–COO⁻), 287.2 (COO–Fe), and 289 eV (COO⁻), which were attributed to the polyacrylic backbone, free carboxyl groups of PAA, and coordination bonds between Fe and carboxyl groups in PAA. After assembly with MNPs, binding energies of carbon atoms in the sulfate ester, guanidine, hydroxyl, and carboxyl groups significantly shifted. Specifically, the COO–Fe peak in MNPs shifted to 288.5 eV, indicating the formation of new coordination interactions between previously uncoordinated Fe ions on MNPs and carboxyl groups of Fu. The COO⁻ and C–OH peaks in F NPs respectively shifted to 289.3 and 284.8 eV, reflecting the formation of hydrogen bonds during the assembly of FM NPs. The C–O/C–N and C=O/C=N peaks of F NPs respectively shifted to 285.7 and 287.7 eV, suggesting the presence of electrostatic interactions between MNPs and the guanidine groups of Prt. Correspondingly, the deconvoluted N 1 s spectrum of FM NPs showed binding energy shifts in the peaks corresponding to C=NH (400.3 eV), O=C–NH⁻ (398.7 eV), C–NH–C (397.6 eV), and C–N (396.9 eV), attributed to guanidine and primary amino groups in Prt. Additionally, the notable shift in the C–N peaks (285.7 eV) was also attributed to the coordination of nitrogen atoms, specifically amino groups in Prt, with Fe to form Fe–N bonds. This was further supported by significant shifts in the deconvoluted Fe^III^ 2p_1/2_ and Fe^III^ 2p_3/2_ peaks in the Fe 2p spectrum and the emergence of a new peak at 398.5 eV (C–NH–Fe) in the N 1 s spectrum of FM NPs. Furthermore, the O 1 s spectrum of FM NPs exhibited significant shifts in the S–O (531.8 eV) and O=S=O (534.6 eV) peaks, while the C 1 s spectrum showed a shift in the C–S peak to 284.1 eV, and the S 2p spectrum revealed shifts in the S 2p_1/2_ (167.9 eV) and S 2p_3/2_ (166.4 eV) peaks. These shifts originated from sulfate ester groups in Fu and were attributed to the reorganization of electrostatic interactions induced by the participation of MNPs in the assembly process. These findings indicated that the surface of the MNP particles could engage in significant intermolecular interactions with Prt, facilitating their co-assembly with Fu to form NPs and achieve a self-assembled stable state. This reconstruction resulted in FM NPs exhibiting a smaller particle size and narrower size distribution (Fig. 2A). More importantly, these newly established and reversible intermolecular forces, including hydrogen bonding, electrostatic interactions, and coordination interactions, drove the aggregation of MNPs within this specific nanoscale environment (Fig. 2C, 2), ultimately leading to a pronounced inversion of MRI T1/T2 signals (Fig. 2E–H).

Gt was utilized to construct the MMP-2/−9-responsive and MRI T1/T2 signal-switchable nanosensor FMG NPs. Beyond providing enzymatic sensitivity, Gt was found to promote further aggregation of MNPs (Fig. 2C, 2), significantly enhancing T2-weighted imaging signals (Fig. 2E, 2). The XPS analysis confirmed that Gt extensively interacted with components of FM NPs through intermolecular forces, which were closely associated with the specific amino acid composition of Gt. The deconvoluted C 1 s spectrum of FMG NPs revealed a new peak at 287.5 eV (pyrrolidino C–N), originating from the high content of proline and hydroxyproline in Gt, while multiple peak shifts at 284.16 (C–C/C=C/C–H), 284.9 (C–S), 285.0 (C–OH), 285.7 (C–COO⁻), 286.0 (C–O/C–N), 288.5 (C=O/C=N), 288.9 (COO–Fe), and 289.5 eV (COO⁻) reflected significant interactions between Gt and FMG NPs. Specifically, the shift in the C–H peaks in the C 1 s spectrum reflected hydrophobic interactions between the 6 C methyl groups of fucose units in Fu and alanine or proline residues in Gt, as well as valine residues in Prt and these same residues in Gt. In addition, shifts in the C–OH, C–N, and C=N peaks in the C 1 s spectrum suggested hydrogen bonding between hydroxyl groups in Fu and guanidine groups in Prt with hydroxyproline and serine residues in Gt, as further supported by shifts in the O 1 s spectrum at C–OH (531.1 eV) and the N 1 s spectrum at C= NH (401.4 eV), O=C–NH⁻ (400.6 eV), and C–NH–C (398.3 eV). Shifts in the peaks of COO–Fe and C–N in the C 1 s spectrum indicated that carboxyl and amino groups in Gt participated in Fe coordination, as evidenced by the shifts in the O 1 s spectrum at COO–Fe (532.4 eV), in the N 1 s spectrum at C–NH–Fe (399.3 eV), and in the Fe 2p spectrum at Fe^III^ 2p_1/2_ (727.5 eV) and Fe^III^ 2p_3/2_ (712.5 eV). Furthermore, shifts in the C–S, C–COO⁻, and COO⁻ peaks in the C 1 s spectrum reflected electrostatic interactions involving positively charged groups in type A Gt, such as guanidine, amino, and pyrrolidino groups. These interactions were corroborated by shifts in the O 1 s spectrum at O=S=O (534.0 eV), S–O (533.7 eV), and COO⁻ (530.1 eV), as well as shifts in the S 2p spectrum at S 2p1/2 (170.2 eV) and S 2p3/2 (168.6 eV), attributed to sulfate ester groups in Fu.

Collectively, as shown in Fig. 3C, these findings suggest that Gt extensively interacts with MNPs, Fu, and Prt through a combination of intermolecular forces, including hydrophobic interactions, hydrogen bonding, coordination, and electrostatic interactions, which collectively drive the assembly of FMG NPs. These interactions not only improve MNP encapsulation efficiency and reshape nanoparticle morphology, but also induce pronounced intermolecular tightening and nano-interfacial reinforcement within the nanostructure, leading to enhanced compaction and sub-particle clustering, in agreement with the reduced particle size and improved uniformity observed in DLS measurements. This structural transformation underlies the dual-mode MRI contrast modulation of FMG NPs, in which T1 suppression arises from water proton shielding and T2 enhancement results from intensified magnetic dipole interactions. Upon enzymatic degradation by Gt-sensitive proteases, these tightly organized architectures are expected to disassemble, disrupting intra- and interparticle interactions and dynamically modulating the surrounding MRI environment, further supporting their responsiveness and adaptability as a precision imaging platform, and illustrating how Gt-mediated compaction and interfacial reinforcement can be harnessed to modulate dual-mode MRI contrast through structural design.

### Enzymatic remodeling enables dynamic MRI signal transformation

The sensitivity of FMG NPs to the MMP-9 protease and their properties as MRI contrast agents were investigated. During the optimization process in Sect. 2.1, the selected FMG NPs demonstrated significant enzymatic sensitivity, while the size distribution of FM NPs lacking Gt remained largely unaffected by MMP-9 (Fig. 4A). This suggested that MMP-9’s sensitivity to FMG NPs was primarily driven by the high proportion of Gt incorporated within them. As shown in Fig. 4B, after 6 h of MMP-9 treatment, the particle size of FMG NPs expanded to the near-micrometer scale (light-gray region), with MNPs also diffusing within this area (dark-gray particles within the light-gray region). This indicated that enzymatic treatment induced significant nanoparticle swelling, compromised the integrity of the outer barrier, and increased interparticle distances of MNPs.

To investigate the ability of FMG NPs to convert environmental MMP-9 into MRI signals, different concentrations of MMP-9 (3–0.03 µg/mL) were tested. As shown in Fig. 4C, both T1- and T2-weighted MRI signals varied with changes in the MMP-9 concentration. T1 signals, initially quenched due to assembly into FMG NPs, gradually recovered with MMP-9 treatment time, while the enhanced T2 signals progressively weakened. This behavior exhibited a dose-dependent response to MMP-9 at most time points (Fig. 4D, 4), indicating that the gradual disassembly of FMG NPs allowed MNPs to return to an unpacked state, effectively reflecting the environmental MMP-9 concentration. These results confirmed that FMG NPs acted as MMP-9-triggered dual-switchable MRI contrast agents. Unlike more-common MRI switchable nanoparticles that transition from a T1 "off state" to an "on state" upon stimulation by enzymes such as hyaluronidase or MMP-2 for tumor imaging [33, 47], or those that switch from both T1 and T2 "off states" to "on states" triggered by glutathione [48], or those that transition from a T1 "off state" and T2 "on state" to both T1 and T2 "on states" triggered by low pH [34], FMG NPs exhibited an initially T1 "off state" and T2 "boosted state," which transformed into a T1 "on state" and T2 "on state" upon exposure to MMP-9. The use of dual signals and their inverse changes offers significant advantages, allowing the elimination of interference from varying target amounts across different tissues, such as thrombi of different ages, by calculating the signal ratio.

The inverse relationship between the two signals amplifies the difference in their ratio. For example, after a 2-h reaction period, the concentration difference between 3 and 0.03 μg/mL of MMP-9 resulted in a 2.1-fold increase in the T1-weighted intensity and an 8.3-fold decrease in the T2-weighted intensity. When analyzed using the T1/T2 or T2/T1 ratio, this difference was further amplified to 16.7-fold (Fig. 4F, 4). These results demonstrated that FMG NPs could reliably convert environmental MMP-9 concentrations into MRI signals, suggesting their potential for further evaluation of thrombus age diagnosis.

### Development of a full-spectrum thrombus aging model for in vivo imaging

**Fig. 5:**
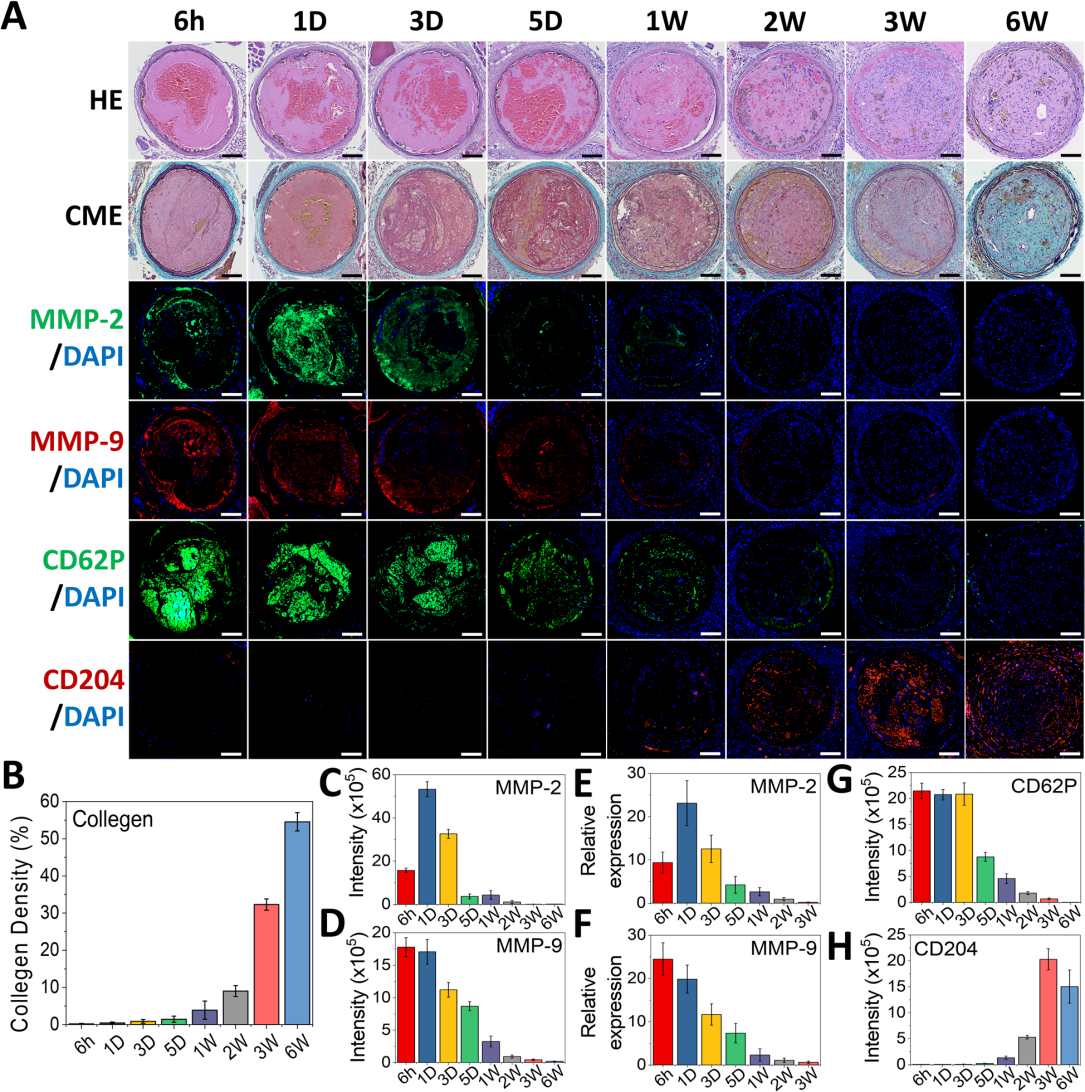
Establishment and characterization of a multi-stage thrombus model. **A** Representative images of hematoxylin–eosin (HE) staining, Masson's trichrome staining, and immunofluorescence staining for MMP-2, MMP-9, CD62P, and CD204 in carotid artery thrombi at different time points. **B**–**D**, **G**, H Quantification of fluorescence intensity using ImageJ software (*n* = 5). **E**, **F** Relative mRNA expression levels of *Mmp2* and *Mmp9* in thrombotic arteries, normalized to *Actb* and presented as fold changes relative to the contralateral uninjured carotid artery (set as 1.0) (*n* = 3). Data are presented as mean ± SD

To date, a comprehensive whole-age thrombus rodent model encompassing all stages of thrombus maturation and organization has not been established. In this study, we aimed to induce thrombus formation by applying FeCl_3_ to vessels and then observe histological changes at consecutive time points from 6 h to 6 weeks (Fig. 5). This model offered several experimental advantages, including consistent thrombus formation, stable thrombus sizes, easy access for analysis, and minimization of intergroup variability. It supported high-throughput blood flow for rapid nanoparticles accumulation, and its high survival rate made it suitable for long-term studies. Based on previously reported clinical data on histological changes associated with human thrombus aging [1–3], this model demonstrated a progressive transformation of thrombi from an initial mixed matrix to a well-organized clot. This transformation included a gradual reduction in red blood cells (HE), a decrease in the platelet content (CD62P), increased infiltration of immune cells (hematoxylin, DAPI, and CD204), and an overall increase in thrombus organization (Fig. 5A). Notably, after 3 weeks, there was a significant rise in the collagen content, increasing from 3.9% at 1 week to 54.6% at 6 weeks (Fig. 5B). Additionally, elastin lost its healthy morphology (wavy lines) after thrombus formation and showed fragmentation after 3 weeks (CME). These histological changes indicated that this whole-age thrombus rodent model can accurately reflect tissue states associated with different thrombus ages.

Consistent with a human specimen analysis [1–3], this model also exhibited high expressions of MMP-2 and MMP-9 during the early to mid-stages of thrombus formation, with expression levels decreasing as the thrombus aged, as confirmed by both the protein and mRNA levels. MMP-2 expression peaked at 1 day, followed by a sharp decline after 3 days (Fig. 5C, 5). In contrast, MMP-9 showed a more-gradual decrease in expression as the thrombus aged (Fig. 5D, 5), suggesting that MMP-9 plays a more-critical role in distinguishing thrombus ages. Additionally, potential targeting markers of FMG NPs, CD62P, and CD204, were evaluated in this model. CD62P remained at a relatively high level within the first 3 days and continued to be detectable at the thrombus site until 2 weeks (Fig. 5G). In contrast, CD204 expression became more apparent at 1 week, peaked at 3 weeks, and then began to decline (Fig. 5H). This pattern aligned with the involvement of CD204-positive macrophages in the thrombus organization process [28, 29]. The complementary expression patterns of CD62P and CD204 along the thrombus aging timeline suggest their potential utility in developing targeted therapies for thrombi of different ages. This full-spectrum thrombus model provides a valuable platform for validating diagnostic tools aimed at identifying thrombus age and remodeling stage.

### In vitro and in vivo thrombus targeting and MRI visualization

**Fig. 6:**
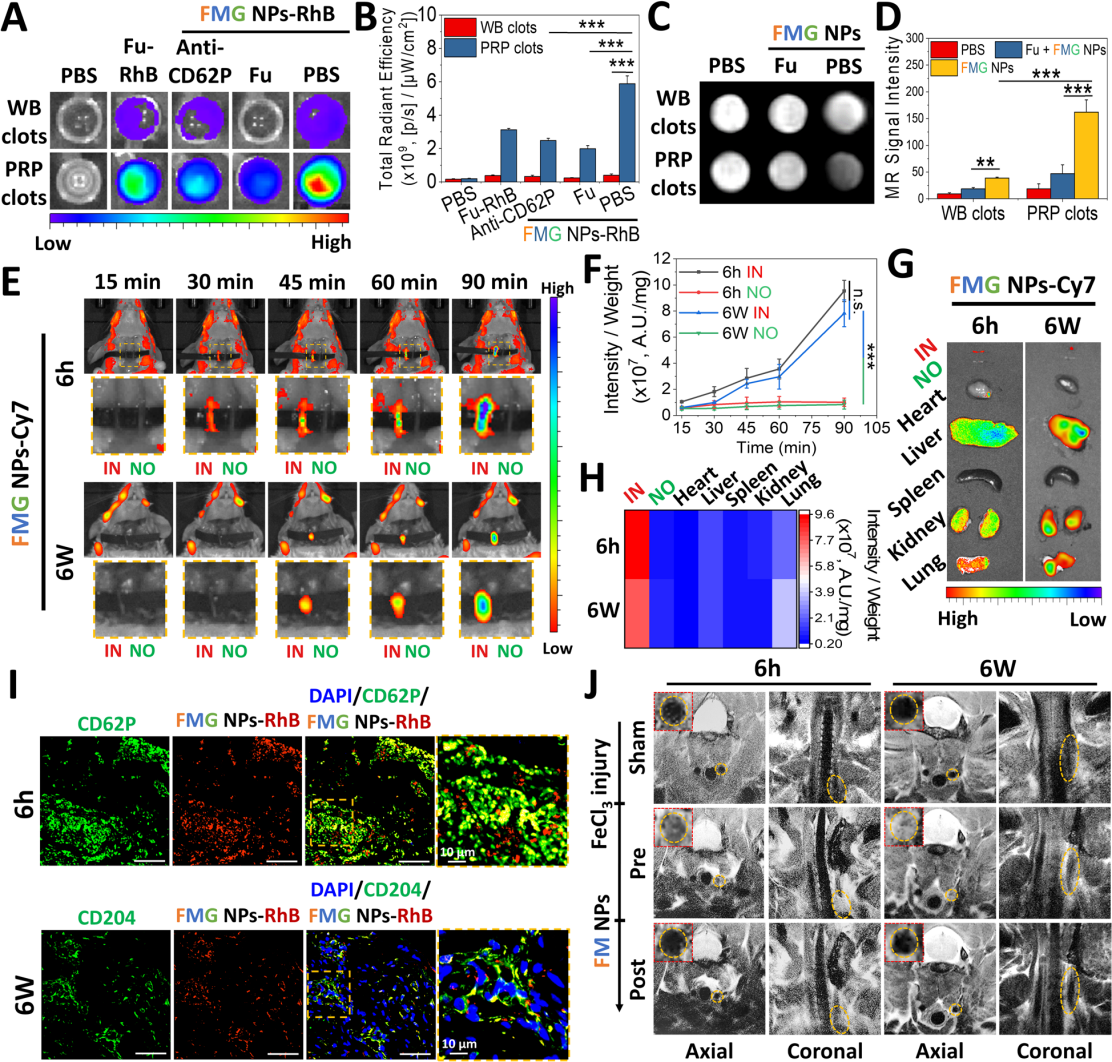
Thrombus-binding affinity, targeting ability, biodistribution, and MR imaging. **A** IVIS images of whole blood and platelet-rich clots treated with Fu or RhB-labeled nanoparticles for 30 min, with or without pre-blocking. **B** Quantitative analysis of the total radiant efficiency from IVIS images (**A**) (*n* = 4). **C** T2-weighted MR images of whole blood and platelet-rich clots treated with Fu or nanoparticles for 30 min, with or without pre-blocking. **D** Quantitative analysis of the MR signal intensity (**C**) (*n* = 4). **E** Targeting ability analysis using a FeCl_3_-induced thrombus model in ICR mice treated with Cy7-labeled FMG NPs via an intravenous injection. IN: injured vessel; NO: normal vessel (control). **F** Quantitative analysis of the total radiant efficiency from IVIS images (E) (*n* = 4). **G** IVIS spectrum images of major organs to assess the biodistribution. **H** Quantitative analysis of the total radiant efficiency from IVIS images (**G**). **I** Co-localization analysis of RhB-labeled FMG NPs with CD62P or both CD62P and CD204 in FeCl_3_-induced thrombus in ICR mice (scale bar: 100 µm). **J** T2-weighted MR images of FM NPs in FeCl_3_-induced thrombi in ICR mice. Data are expressed as mean ± SD. Statistical significance was assessed using Student’s t-test (n.s., non-significant difference, *** *p* < 0.001)

To evaluate the thrombus-targeting capability of FMG NPs, in vitro whole-blood (WB) clots were prepared using rat tail arterial blood, and platelet-rich plasma (PRP) clots were prepared using PRP separated from whole blood. These two types of thrombi were used to compare the binding ability of the nanoparticles to thrombi with different levels of activated platelets. Fu was labeled with the fluorescent molecule rhodamine B (RhB) to facilitate observation. As shown in Fig. 6A and 5B, FMG NPs-RhB demonstrated significant thrombus-binding capability in both WB clots and PRP clots, with respective binding values of 4.02 × 10^8^ and 5.88 × 10^9^ [p/s]/[μW/cm^2^]. The binding of FMG NPs-RhB to PRP clots was approximately 14.6-times higher than that to WB clots. Furthermore, pre-blocking with either a CD62P antibody (anti-CD62P) or Fu significantly reduced the thrombus-binding capability, indicating that FMG NPs bound to activated platelets through interactions between surface Fu and CD62P. In T2-weighted MRI, thrombi that initially appeared as low signals (white) exhibited high signals (dark) after treatment with FMG NPs (Fig. 6C). This treatment enhanced the T2-weighted signal intensity of PRP clots by approximately 8.66-fold (Fig. 6D). These findings suggested that FMG NPs exhibited excellent thrombus-binding capability as a targeted delivery vehicle and provided substantial contrast enhancement as an MRI agent.

After confirming the surface bioactivity and imaging capability of FMG NPs, the fluorescent molecule, cyamine7 amine (Cy7), was conjugated to Fu, forming FMG NPs-Cy7. These nanoparticles were administered via a tail vein injection, and their targeting capability and biodistribution were assessed using the rodent model established in Sect. 2.3. As shown in Fig. 6E and 5 F, NP accumulation at a fresh thrombus site (formed 6 h prior, injury (IN) site) was detectable within 30 min, and the accumulation continued to increase over time. By 90 min, the accumulation of FMG NPs-Cy7 at the IN site was 11.7-times higher than at a normal vessel (normal (NO) site) (Fig. 6F). For older thrombi formed 6 weeks earlier, FMG NPs-Cy7 signals were detectable at the IN site within 45 min, and by 90 min, the accumulation at the IN site was 9.4-times higher than at the NO site. Among major organs, FMG NPs-Cy7 showed the highest accumulation at the IN site (weight-adjusted signal intensity), which was 4.54-times higher than that in the lungs, the organ with the second-highest accumulation (Fig. 6G, 6). In Fig. 6I, significant FMG NPs-Cy7 signals were observed for both 6-h and 6-week thrombi. These signals respectively co-localized well with CD62P and CD204 in 6-h and 6-week thrombi, as indicated by the yellow regions. These results suggested that FMG NPs can rapidly target thrombus sites through their high binding affinity to CD62P and CD204, regardless of the thrombus age. Previous studies using Fu-based nanoparticles for managing thrombosis demonstrated similar targeting efficiencies [24, 44]. This consistency underscores the excellent targeting efficiency and reproducibility of Fu in thrombus targeting. Moreover, inspired by the involvement of CD204-positive macrophages in the reprogramming process during thrombus organization and aging, this study discovered for the first time that Fu can target organized thrombi. This finding could significantly contribute to the development of thrombus-treating drugs and expands the application scope of Fu.

In Fig. 6J, the MRI capability of this nanoformulation was evaluated through a tail vein injection of FM NPs, which due to their lack of an MMP-2/9-response capability, can maintain a T2 "boost"/T1 "off" state. After 2 h, significant signal enhancement in T2-weighted images (appearing darker) was observed at both 6-h and 6-week thrombus sites (indicated by yellow-orange circles). This demonstrated that the accumulation of this nanoformulation at thrombus sites effectively altered the magnetic field environment, producing significant MRI contrast effects.

### Quantitative thrombus staging via MRI signal ratio dynamics

**Fig. 7:**
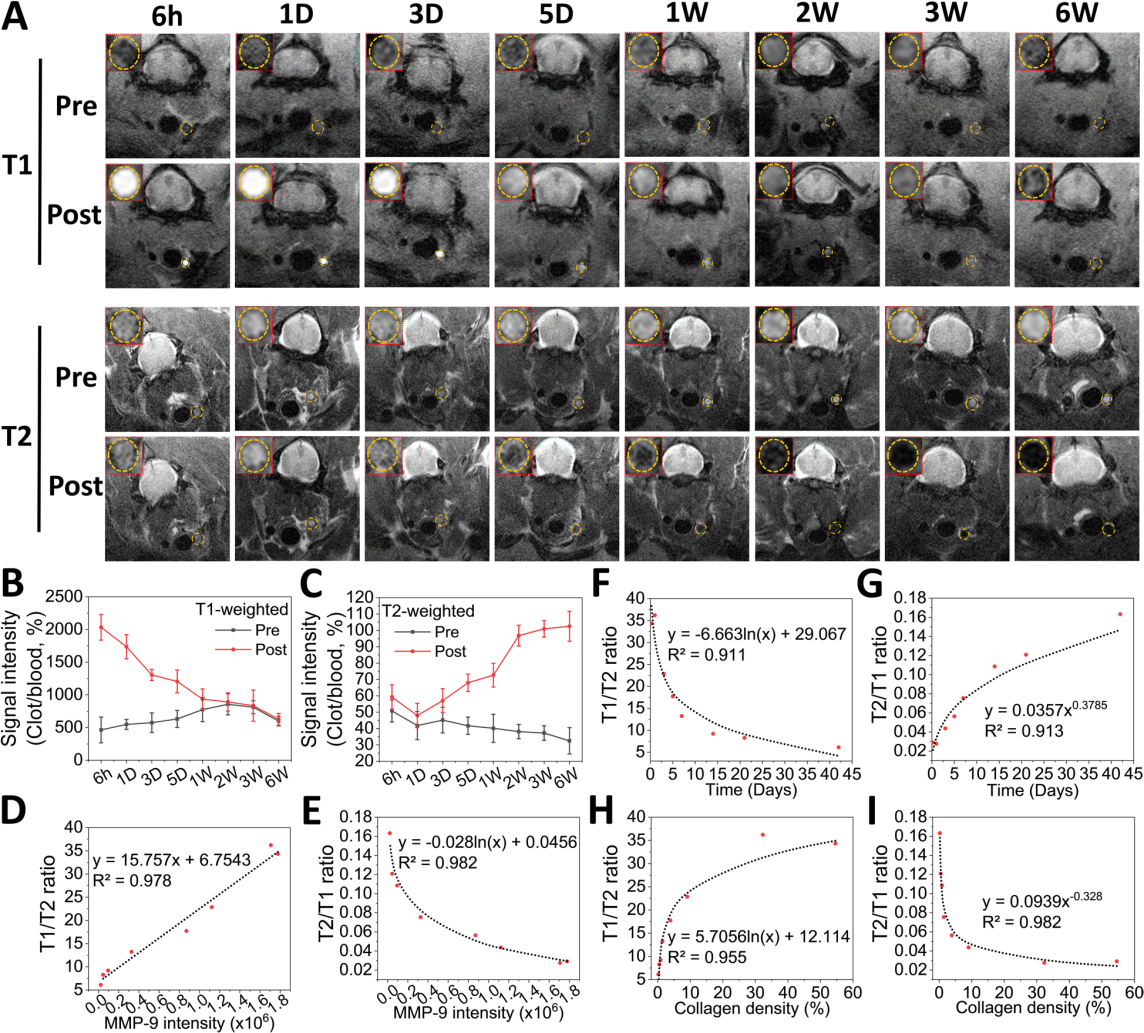
Thrombus age diagnosis. **A** T1- and T2-weighted MRI of FMG NPs in FeCl_3_-induced carotid artery thrombi in ICR mice. Pre, different time periods (6 h to 6 weeks) after 3 min of FeCl_3_ injury. Post, 2 h after introduction of FMG NPs. **B–I** Corresponding quantitative analyses and calculations (*n* = 3). Data are expressed as mean ± SD

To investigate the MMP-2/9-triggered MRI switching capability of FMG NPs in diagnosing thrombus ages, mice with thrombi of different ages (Sect. 2.4) were administered FMG NPs via a tail vein injection. MRI was performed 2 h post-injection. In Fig. 7A, for 6-h thrombi with high MMP-2/9 expression, the incorporation of Gt (FMG NPs) resulted in negligible signal enhancement in T2-weighted images, in contrast to the noticeable enhancement observed with FM NPs (Fig. 6J). However, T1-weighted images showed a significant enhancement of approximately 4.4-fold compared to the untreated group (Pre) (Fig. 7B). Additionally, the T2-weighted image signal enhancements for FMG NPs and FM NPs in 6-week thrombi were similar, showing an increase of approximately 3.2-fold compared to the untreated group (Pre) (Fig. 7C). These observations of thrombi at 6 h and 6 weeks demonstrated that the MMP-2/9-triggered MRI switching capability imparted by Gt to FMG NPs (Fig. 4) was effectively exhibited in vivo. Upon collecting T1- and T2-weighted image signals of FMG NPs from thrombi of various ages, it was observed that T1 signals significantly decreased as the thrombus aged, with the decline slowing after the thrombus age had exceeded 1 week (Fig. 7B). Conversely, T2 signals notably increased after 1 day and continued to rise with thrombus age until the increase slowed after 2 weeks (Fig. 7C). This pattern was correlated with MMP-9 expression levels at different thrombus ages (Fig. 5D, 5), suggesting that the MRI switching capability of FMG NPs maintained a dose-dependent relationship with MMP-9 expression in vivo within thrombi. FMG NPs combined with conventional MRI scanning techniques (T1: FISP; T2: SE-highres) significantly improved signal detection. Specifically, T1-weighted images showed a 3.3-fold decrease in signal intensity between 6 h and 6 weeks (Fig. 7B), while T2-weighted images demonstrated a 2.2-fold increase in signal intensity between 1 day and 6 weeks (Fig. 7C). Furthermore, when calculating T1/T2 or T2/T1 ratios, signal intensities changed by 6.6-fold (Fig. 7F, 7).

Compared to previous methods that attempted to detect thrombus ages using specialized MRI techniques and US elastography, this study demonstrated more-significant temporal changes [1, 12, 49] (Table S2). For example, black-blood MRI, which relies on blood flow suppression to enhance thrombus contrast, is effective for detecting acute thrombi but has limitations in assessing chronic thrombi (> 4 weeks) [1, 14]. As fibrin remodeling progresses, chronic thrombi become less permeable to surrounding fluids, reducing the contrast in black-blood MRI and making it difficult to distinguish stable chronic thrombi from resolving thrombi [15]. Additionally, black-blood MRI lacks biological markers to provide a quantitative assessment of thrombus degradation over time. In contrast, FMG NPs leverage MMP-2/9 responsiveness to dynamically modulate the T1/T2 MRI signal, enabling precise thrombus age differentiation with quantitative accuracy. Similarly, ultrasound elastography, which estimates thrombus stiffness based on mechanical properties, has been explored for thrombus age assessment but remains limited in several aspects. Its accuracy is highly dependent on probe placement and operator expertise, leading to variability in measurements [15, 16]. Moreover, ultrasound elastography performs well in superficial thrombi but has reduced sensitivity for deep vein thrombi (DVT) and pulmonary embolism (PE) due to signal attenuation. FMG NPs, in contrast, provide a standardized, operator-independent MRI readout that remains consistent across different thrombus locations, making them a more reliable tool for clinical thrombus age diagnosis. Additionally, thrombi in this study were much smaller than those used in previous animal models, highlighting the potential of FMG NPs for clinical diagnosis of small thrombi [1, 12, 49]. A correlation analysis between MMP-9 expression and the T1/T2 or T2/T1 ratio revealed a strong linear relationship between the T1/T2 ratio and MMP-9 expression in thrombi (*R*^2^ = 0.978), and a power-law relationship for the T2/T1 ratio (*R*^2^ = 0.982) (Fig. 7D, 7). When analyzing the correlation with thrombus age (in days), the T1/T2 and T2/T1 ratios respectively exhibited logarithmic (*R*^2^ = 0.911) and power-law relationships (*R*^2^ = 0.913) (Fig. 7F, 7). Additionally, when correlated with the degree of organization (collagen density), the T1/T2 ratio showed a logarithmic relationship (*R*^2^ = 0.955) and the T2/T1 ratio showed a power-law relationship (*R*^2^ = 0.982) (Fig. 7H, 7). These highly significant correlations indicated that FMG NPs could effectively convert information regarding thrombus age and degree of organization into MRI signals, demonstrating their potential as a foundation for standardized thrombus staging tools in clinical decision-making and imaging-based therapeutic stratification. Collectively, these findings demonstrate for the first time that a nanoprobe can transform thrombus maturation signatures into quantifiable dual-mode MRI signals across the full age spectrum in vivo.

### Enzyme-triggered fibrinolytic drug release and in vitro thrombolysis

Uk is a commonly used thrombolytic agent, but its short half-life and systemic hemorrhagic side-effects highlight the need for a targeted, controlled-release delivery system [43–46]. Previous research on fibrinolytic enzyme-targeted nano-delivery systems explored various environment-sensitive and responsive release strategies to enhance delivery precision and reduce hemorrhagic side-effects in other organs. These methods include US- [43], shear stress- [44], near infrared (NIR)- [43, 45], and reactive oxygen species (ROS)-stimulated [46] release, all of which have been widely studied. While photothermal and US bubble-responsive releases offer high precision and therapeutic assistance, their limited penetration depth and requirement for specific equipment restrict their practical application. In contrast, ROS-responsive release is not limited by the same constraints but has relatively low precision, as ROS are produced by a wider range of cell types due to their generalized role in cellular metabolism and signaling [50]. Enzyme stimuli, being relatively mild and highly selective for specific substrates, allow construction of enzyme-responsive nanomaterials with precise targeting abilities for specific lesions [51]. Given the high expressions of MMP-2/−9 at thrombus sites (Fig. 5), this study is the first to investigate the use of MMPs to precisely deliver fibrinolytic drugs for treating thrombi.

**Fig. 8:**
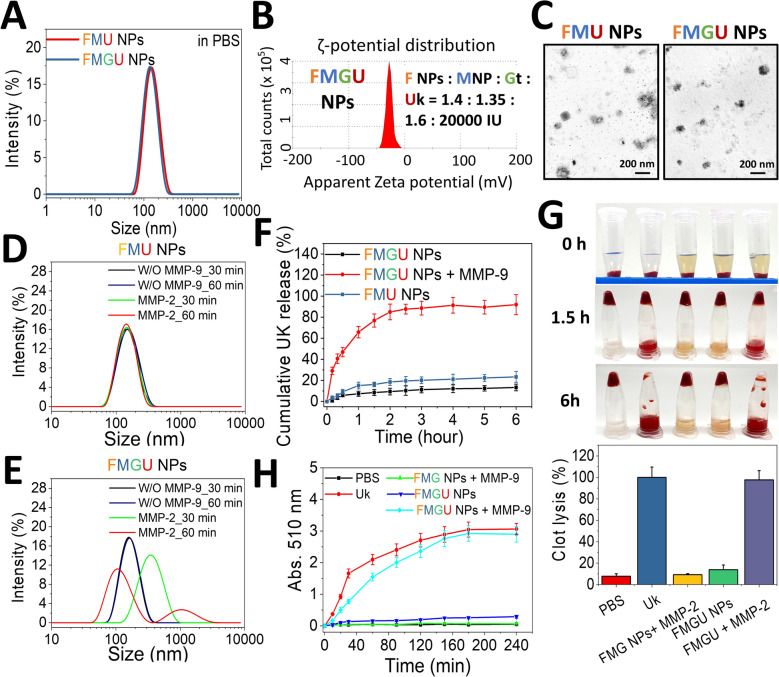
Uk loading, MMP-responsive drug release, and thrombolysis in vitro. **A** Particle size distribution. **B** ζ-potential distribution. **C** TEM images. **D**, **E** Size distribution of FMU NPs and FMGU NPs in pH 6.8 PBS at 37 °C, with or without MMP-9 (0.75 µg/mL). **F** Cumulative release of UK from FMGU NPs in pH 6.8 PBS at 37 °C, with or without MMP-9 (0.75 µg/mL MMP-9) (*n* = 3). **G** Photographs of 100 µL whole-blood clots under different treatments, incubated at 37 °C (0.75 µg/mL MMP-9; 2400 IU/clot Uk) with clot lysis measured by the weight of the remaining clot after 6 h (*n* = 3). **H** Thrombolytic kinetic profile of FMGU NPs measured by a halo-plate assay at 37 °C with or without MMP-9 (2400 IU Uk/clot) (*n* = 8). Data are expressed as mean ± SD

To address this issue, the nanoparticles established in this study, were deemed suitable for precisely delivering Uk to thrombus sites and achieving controlled release. This was accomplished through their thrombus-targeting ability and sensitivity to MMP-2/−9, thereby reducing side-effects and dosage requirements. In Figure S3A, regardless of the Gt ratio, loading different amounts of Uk slightly reduced the average particle size to between 130 and 155 nm. Additionally, the previously observed size variation pattern with changing Gt content disappeared, showing no discernible pattern. The surface potential also became less negative with an increasing Uk content (Figure S3C). These phenomena were likely due to competitive electrostatic interactions between the positively charged Uk (with pI values of ca. 9.4) and Gt with Fu or MNPs. However, when Uk loading was increased to 40,000 IU, the particle size increased to approximately 169.2 nm, and after 16 h, it had further increased to 331 nm, with the PDI rising to 0.3 (Figure S3F). Such increases would be detrimental to Uk delivery, as the instability of off-target nanoparticles over time could result in free Uk being dispersed into the circulation. In contrast, with a weight ratio of F NPs: MNP: Gt: Uk of 1.4 mg: 1.35 mg: 1.6 mg: 20,000 IU, forming FMGU NPs, stability was well maintained under physiological conditions (Figure S3G). These FMGU NPs had an average particle size of 133.7 nm and a PDI of approximately 0.13 (Table S1), with a narrow, monodispersed size distribution (Fig. 8A). The surface charge was about − 13.5 mV (Fig. 8B), and morphologically, they were indistinguishable from FMG NPs (Fig. 8C). Additionally, as a control group, FMU NPs without Gt were fabricated. These nanoparticles exhibited a similar particle size and PDI to FMGU NPs (Table S1). However, consistent with findings from Sect. 2.1, the absence of Gt resulted in a more-negative surface potential for FMU NPs, of approximately − 26.4 mV (Table S1).

FMU NPs maintained a consistent particle size distribution in both the presence and absence of MMP-9 (Fig. 8D). However, FMGU NPs showed a broadened and right-shifted particle size distribution after 30 min in the presence of MMP-9, and a multimodal distribution after 60 min (Fig. 8E). This indicated that the loading of Uk did not affect the enzyme sensitivity of this nanoformulation. Additionally, Uk was rapidly released during particle degradation, with approximately 90% of Uk being released within 2 h. In contrast, only about 10%–15% of Uk was released from FMU NPs or FMGU NPs in the absence of MMP-9 (Fig. 8F). The thrombolytic activity of the released Uk was evaluated using in vitro rat thrombi. In Fig. 8G, after treatment with Uk or nanoparticles (equivalent to 2400 IU/clot) and co-incubation for 1.5 h, both the Uk and FMGU NPs with MMP-9 groups showed partial thrombus lysis. After 360 min, the thrombi had been completely lysed. Quantitative analysis of the remaining thrombi revealed that only about 15% of the thrombus had been lysed in the FMGU NP group without MMP-9. In contrast, when MMP-9 was present, thrombus lysis reached a comparable level to that of Uk alone. This demonstrated that FMGU NPs could effectively release Uk in response to MMP-9 and maintain the thrombolytic activity of Uk. Additionally, continuous measurements using a halo-plate assay revealed that although the thrombolytic effect of the FMGU NP + MMP-9 group was slightly inferior to that of Uk within the first 60 min, it achieved comparable thrombolytic effects to Uk after 120 min (Fig. 8H). These results indicated that the enzyme sensitivity of this nanoformulation could effectively be utilized for the loading and release of fibrinolytic agents, resulting in efficient thrombus dissolution.

### In vivo thrombolytic efficacy and hemorrhagic safety evaluation

**Fig. 9:**
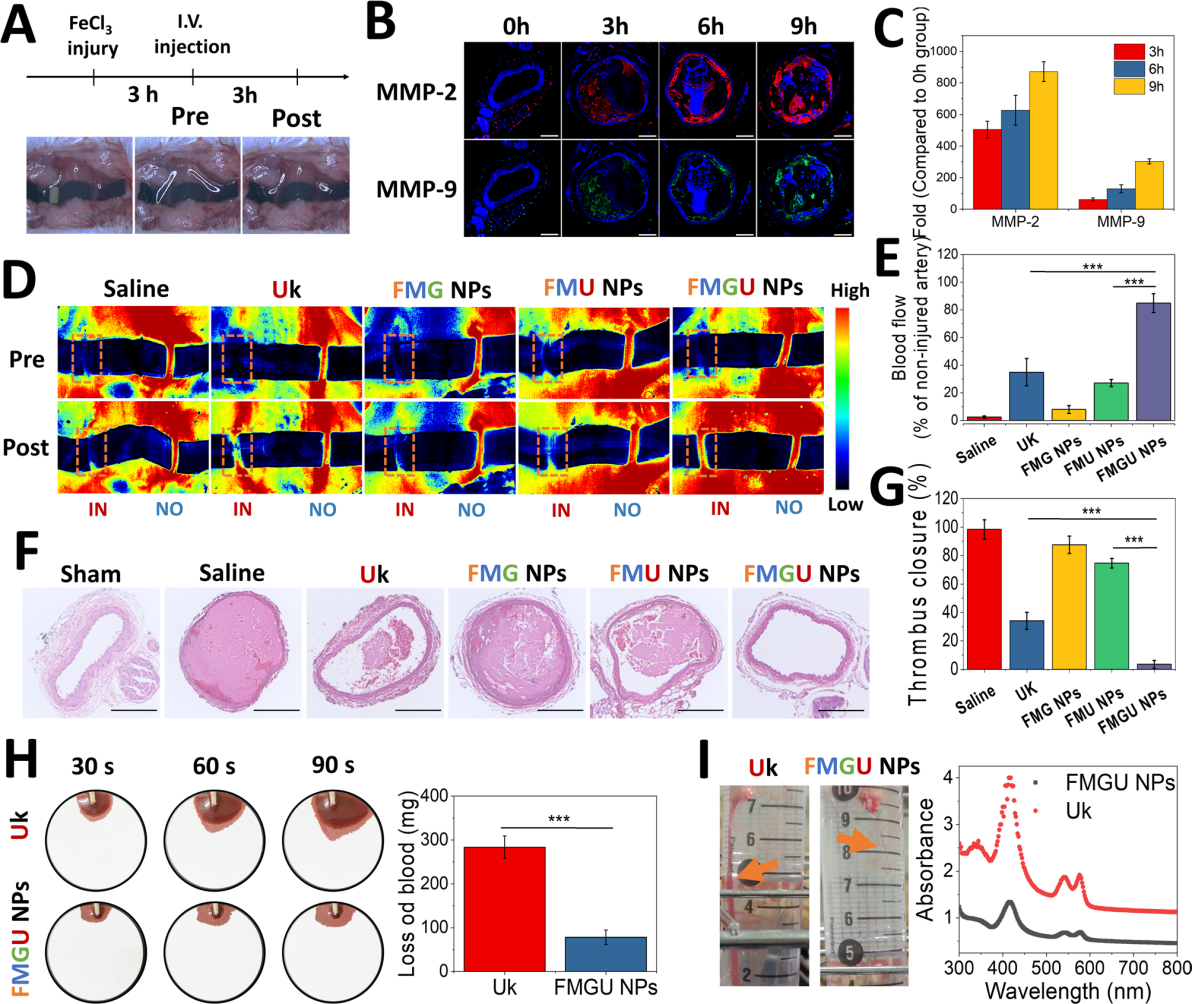
MMP-driven thrombolytic activity in vivo. **A** Schematic representation of the experimental procedure. **B** Immunohistochemical images showing expressions of MMP-2 and MMP-9 at 3 h post-thrombosis induction. **C** Quantitative analysis of MMP-2 and MMP-9 expressions based on (**B**) ImageJ software (*n* = 4). **D** Doppler blood flow images of the FeCl_3_-induced thrombosis model in ICR mice. Orange boxes indicate regions selected for Doppler blood flow analysis. **E** Quantitative analysis of blood flow recovery in (**D**) following administration of Uk or FMGU NPs (64,102 IU Uk/kg BW) (*n* = 4). IN: injured vessel; NO: normal vessel (control). **F** Hematoxylin and eosin (HE) staining of arterial sections at the thrombus site (scale bar = 100 µm). **G** Quantitative analysis of the thrombus area based on (**F**) using ImageJ software (*n* = 4). **H** Tail-bleeding assay in SD rats with FeCl_3_-induced carotid artery thrombosis. After administering Uk or FMGU NPs (64,102 IU Uk/kg BW) for 1 h, filter paper was used to absorb blood from the bleeding site, and the weight difference was recorded for a quantitative analysis (*n* = 3). **I** The tail-bleeding extent assessed after the same treatment as in (**H**). Rat tails were submerged in 37 °C PBS for 30 min, and the hemoglobin content in the PBS solution was quantified by measuring the absorbance spectra of hemoglobin. Data are expressed as mean ± SD. Statistical significance was assessed using Student’s t-test (***p < 0.001)

Fibrinolytic medicine exhibits the highest thrombolytic efficiency against fresh thrombi. Therefore, thrombi at this stage are the most suitable model for evaluating the efficacy of Uk delivery carriers. Following induction of thrombus formation by FeCl_3_ for 3 min and a subsequent waiting period of 3 h, FMGU NPs were introduced to mice via a tail vein injection. After another 3 h, both carotid arteries were exposed using black strips to facilitate monitoring with a laser Doppler flowmeter (Fig. 9A). During this period, MMP-2/9 expression levels were also monitored. Compared to pre-thrombus induction levels, MMP-2/9 expressions in the vascular lumen were significantly elevated, showing a 500–850-fold rise at 3 to 9 h (Fig. 9B, 9), favoring the specific release of Uk at the thrombus site. In Fig. 9D, treatment with FMGU NPs (equiv. to 64,102 IU Uk/kg body weight (BW)) resulted in the most significant blood flow restoration, of approximately 84.9%, compared to 34.9% with Uk and 27.1% with non-responsive FMU NPs (Fig. 9E). This substantial difference was attributed to the thrombus-targeting capability (Fig. 6) and responsive release ability (Fig. 8) of this nanoformulation. A histological analysis and quantification revealed a similar trend. Compared to Uk, FMGU NPs further reduced thrombus closure by 9.7-fold (Fig. 9F, 9). FMGU NPs integrate recent advances in responsive release strategies for Uk-targeted delivery nanoformulations. They demonstrate a high Uk-loading capacity (Table S3), exceptional thrombus-targeting capability (Fig. 6), and strong sensitivity to MMPs (Fig. 7). These features contribute to their remarkable thrombolytic efficiency, significantly surpassing other agents employing different release mechanisms [43–46]. Specifically, FMGU NPs were about 2.6-times more effective than NIR-driven nanoparticles and approximately 5.9-times more effective than H_2_O_2_-triggered nanoparticles, which also do not require external supporting devices (Table S3). Additionally, FMGU NPs were tested against relatively older thrombi (180 min), which were more stable and presented a greater challenge, further highlighting their superior efficacy compared to FMU NPs. Although comparisons with other studies on Uk delivery systems were not rigorous due to differences in thrombus models, targeting efficiency, and sampling times, the overall positive results suggested that proteases, such as MMP-2 and MMP-9, which are highly expressed at thrombus sites have significant potential for developing thrombus-sensitive, specific, and responsive nanocarriers, which could be highly effective for drug delivery or lesion diagnosis applications.

A rodent tail-bleeding assay was performed to assess the hemorrhagic risk associated with FMGU NPs (Fig. 9H, 9). The bleeding volume, a key indicator of hemorrhagic risk, was significantly lower in the FMGU NP-treated group compared to the free Uk group (Fig. 9H). Free Uk treatment (64,102 IU Uk/kg BW) markedly impaired natural hemostasis (orange arrow), resulting in a 2.6-fold higher hemoglobin absorbance at 30 min post-tail amputation, indicating significantly greater bleeding (F9g. 9I). In contrast, FMGU NPs demonstrated a reduced hemorrhagic risk, attributed to their Fu-based thrombus targeting and MMP-2/9 responsiveness. These properties enabled localized and controlled Uk release at thrombotic sites, improving thrombolytic efficacy while minimizing off-target drug release. As a result, FMGU NPs effectively mitigated hemorrhagic complications in non-thrombotic tissues. These findings highlight the potential of FMGU NPs to address the clinical challenge of massive hemorrhaging associated with uncontrolled thrombolytic drug release.

Both FMG NPs and FMGU NPs exhibited excellent blood compatibility, with a hemolysis ratio of < 1% (Fig. 9A). Weekly injections of FMG NPs or FMGU NPs for four consecutive weeks caused no significant damage to major organs (Fig. 9C). Plasma concentrations of liver function markers (alanine aminotransferase (ALT), aspartate aminotransferase (AST), alkaline phosphatase (ALP), and lactate dehydrogenase (LDH)) and kidney function markers (blood urea nitrogen (BUN) and creatinine (CREA)) showed no significant differences between the saline group and the NP-treated group (Fig. 9D), indicating the absence of pathological damage. To further evaluate in vivo clearance, Fe and S contents in major organs and the thrombosed vessel segment were quantified by ICP-MS/MS at 4 weeks post-injection (Fig. 9E). Fe and S levels in major organs were comparable across all groups and remained within normal physiological ranges, suggesting minimal long-term accumulation of MNPs at the imaging dose. Notably, the FMGU NP group showed markedly reduced Fe levels in the thrombosed vessel compared to the saline and FMG groups, suggesting effective thrombus removal. Furthermore, neither FMG NPs nor FMGU NPs exhibited significant cytotoxicity toward human umbilical vein endothelial cells (HUVECs) at a concentration of 1 mg NPs/mL (Fig. 9F). Collectively, these results demonstrated the good biocompatibility of this nanoformulation.

**Fig. 10:**
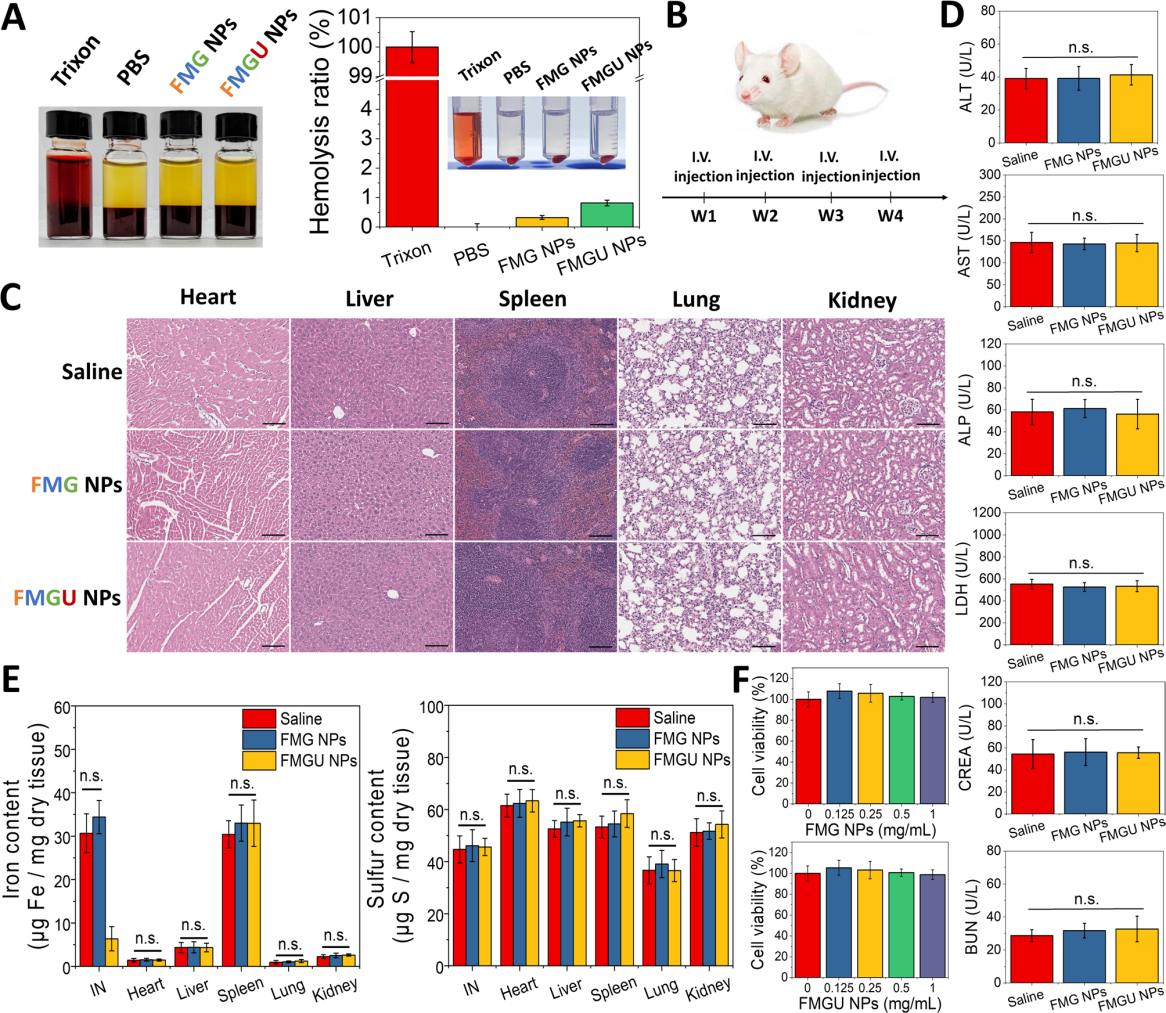
Biocompatibility of FMG NPs and FMGU NPs. **A** Hemolysis assay. Photograph of the mixed solution of whole blood and NPs after 3 h (left) and after centrifugation (right) (*n* = 4). **B** Schematic diagram of the experimental procedure. **C** Soft-tissue sections treated with FMG NPs or FMGU NPs, then stained with H&E (scale = 100 µm). **D** Assessment of liver function markers including ALT, AST, ALP, and LDH, as well as kidney function markers BUN and CREA to evaluate the biocompatibility (*n* = 4). **E** Elemental analysis of Fe and S contents in major organs and the thrombosed carotid artery segment in the FeCl_3_-induced carotid thrombosis mouse model at 4 weeks post-injection. Fe and S were selected as representative elemental markers for MNPs and Fu, respectively. Mice were intravenously injected with FMG or FMGU NPs at doses of 5.6 mg MNP/kg and 3.3 mg fucoidan/kg (*n* = 3). IN, injured vessel. **F** Cell viability assay in HUVECs after 24 h of co-incubation with FMG NPs or FMGU NPs (*n* = 6). Data are expressed as mean ± SD. Statistical significance was assessed using Student’s t-test (n.s., non-significant difference)

## Conclusions

This study presents FMG NPs as a breakthrough in thrombus diagnosis, enabling precise age determination through dual-switchable MRI contrast capabilities responsive to MMP-2 and MMP-9 activities. To our knowledge, this is the first demonstration of a nanoplatform capable of converting thrombus maturation signatures into quantifiable MRI signals across the full spectrum of thrombus age in vivo. By integrating Gt-modulated structural adaptation, FMG NPs not only translated MMP-2/9 activity into distinct T1 and T2 MRI signals but also enhanced signal contrast through nanoprobe compaction and increased MNP clustering, significantly amplifying T2-weighted MRI contrast. The MRI contrast strongly correlated with thrombus maturation markers such as MMP-9 expression, thrombus age, and collagen density, establishing FMG NPs as an advanced imaging platform for thrombus characterization.

Compared to conventional non-invasive diagnostic techniques, such as US elastography and black-blood MRI sequences, FMG NPs demonstrated superior signal contrast, deeper tissue penetration, and greater differentiation between fresh and aged thrombi, without requiring specialized hardware or computational post-processing. These advancements address critical limitations in current thrombus imaging, particularly in diagnosing small-vessel thrombi and differentiating maturation stages. Furthermore, FMG NPs exhibit strong potential as a carrier for targeted fibrinolytic drug delivery. FMGU NPs further enhanced thrombolytic efficiency and specificity through Fu-mediated thrombus targeting and MMP-2/9 responsiveness, improving thrombolysis while minimizing hemorrhagic risks.

Beyond thrombus imaging, Gt played a crucial role in modulating MRI contrast properties by enhancing nanostructural condensation and sub-particle clustering, offering a novel strategy for optimizing nanoparticle-based imaging agents. These structural innovations not only position Gt as a key modulator in MRI nanoformulations but also expand its potential in biomedical imaging and theranostics. By integrating precision imaging and targeted therapy, FMG NPs offer a clinically translatable platform for thrombus diagnosis and personalized thrombolytic intervention, enabling patient-specific treatment strategies tailored to thrombus composition and progression. Furthermore, this study highlights a structurally tunable nanoplatform that couples enzyme-responsiveness with dual-mode MRI signal transformation, laying a materials-driven foundation for pathology-adaptive theranostic systems. The ability of FMG NPs to differentiate thrombus maturation stages also lays the foundation for AI-assisted thrombus classification, supporting data-driven thrombus research and predictive modeling. Collectively, this work not only advances nanoparticle-based MRI strategies but also establishes a clinically translatable paradigm for personalized, pathology-adaptive imaging-guided intervention in thrombus-associated disease.

## Supplementary Information

Supplementary material 1

## Data Availability

The data that support the findings of this study are available from the corresponding author upon reasonable request.
