# Supplementary material for: Programmable MRI contrast switching for spatiotemporal mapping of thrombus maturation via enzyme-directed nanoprobe reconfiguration
Source: Nano Converg. 2025 Oct 30;12:53. doi: 10.1186/s40580-025-00518-w (PMC12575922; doi:10.1186/s40580-025-00518-w)
Supplement: Supplementary file 1 — Supplementary material 1 [file 40580_2025_518_MOESM1_ESM.docx]

Supporting Information (SI)

**Programmable MRI Contrast Switching for Spatiotemporal Mapping of Thrombus Maturation via Enzyme-Directed Nanoprobe Reconfiguration**

Chi Lin ^1, 2^, Fang-Yu Hsu ^1^, Chun-Ming Shih ^3, 4^, Tsai-Mu Cheng ^3, 5^, Alexander TH Wu ^3, 5^, Chia-Hsiung Cheng ^1, 2, 3^, Hsin-Ying Lu ^3, 6^, Chun-Che Shih ^3, 6, 7, *^ and Fwu-Long Mi ^1, 2, 3, 8, *^

^1^ Department of Biochemistry and Molecular Cell Biology, School of Medicine, College of Medicine, Taipei Medical University, Taipei 11031, Taiwan

^2^ Graduate Institute of Medical Sciences, College of Medicine, Taipei Medical University, Taipei 11031, Taiwan

^3^ Taipei Heart Institute, Taipei Medical University, Taipei 11031, Taiwan

^4^ Division of Cardiology and Cardiovascular Research Center, Taipei Medical University Hospital, Taipei 11031, Taiwan

^5^ The PhD Program for Translational Medicine, College of Medical Science and Technology, Taipei Medical University, Taipei 11031, Taiwan

^6^ Division of Cardiovascular Surgery, Department of Surgery, Wan Fang Hospital, Taipei Medical University, Taipei 11031, Taiwan

^7^ Department of Surgery, School of Medicine, College of Medicine, Taipei Medical University, Taipei 11031, Taiwan

^8^ Graduate Institute of Nanomedicine and Medical Engineering, College of Biomedical Engineering, Taipei Medical University, Taipei 11031, Taiwan

* Corresponding authors.

*E-mail addresses:* ccshih0603@tmu.edu.tw (C.-C. Shih), flmi530326@tmu.edu.tw (F.-L. Mi).

*Present address:* No.252, Wuxing St., Taipei 11031, Taiwan.


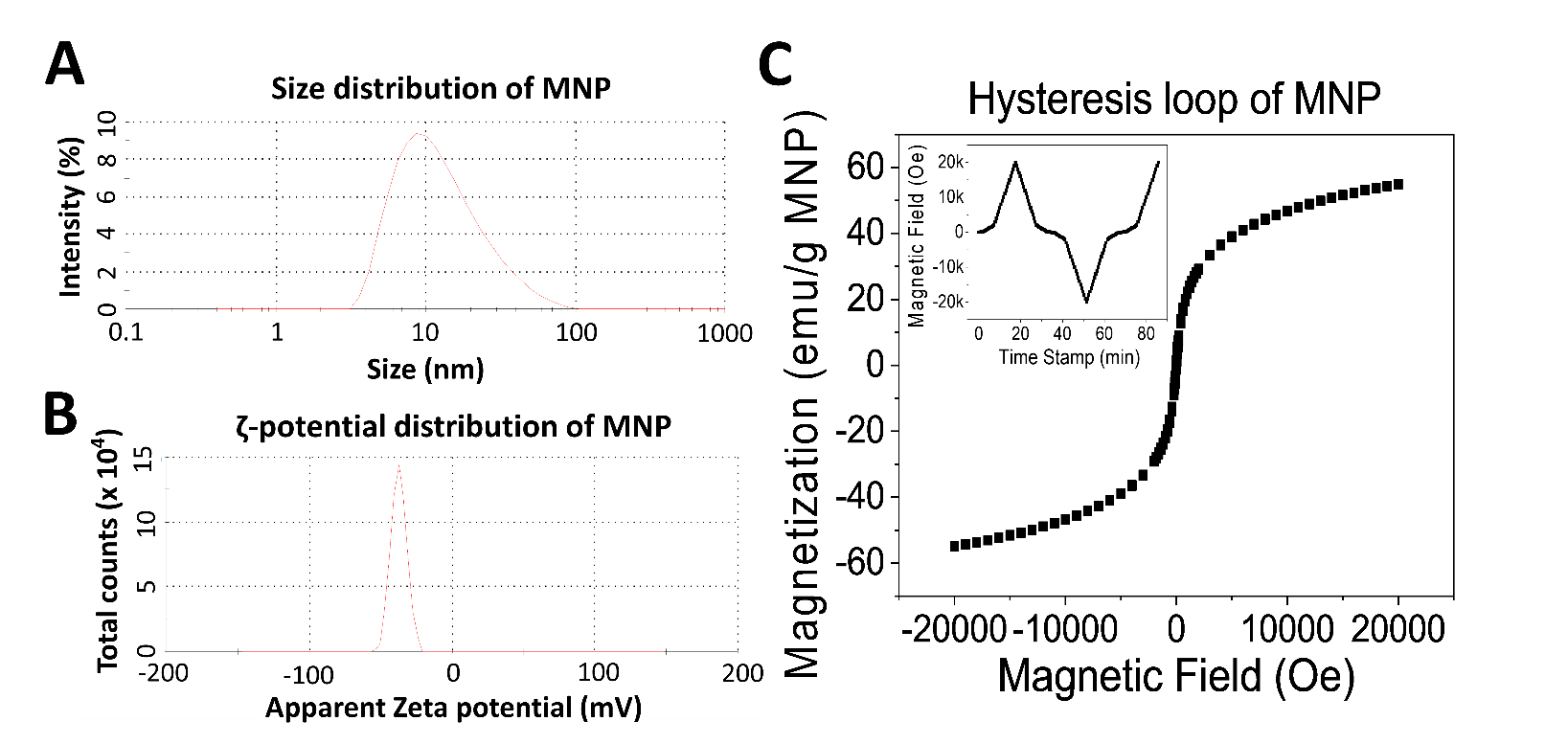


**Figure S1** Preparation and characterization of MNP. (A) Particle size distribution. (B) ζ-potential distribution. (C) Relaxation curve analysis.


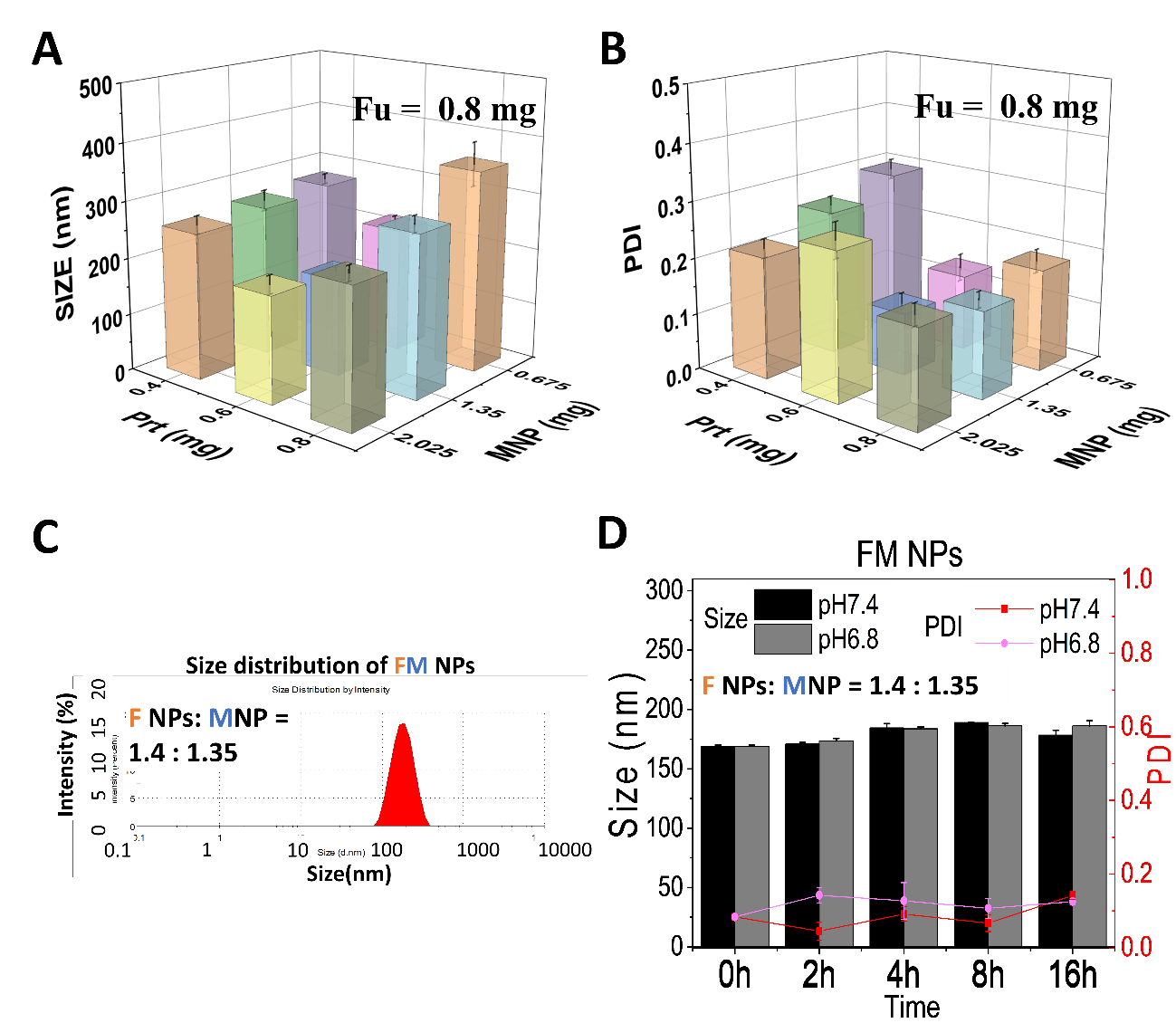


**Figure S2** Preparation and optimization of FM NPs. **(**A) Mean particle size (*n*=3). (B) Polydispersity Index (PDI) (*n*=3). (C) Size and ζ-potential distribution (*n*=3). (D) Stability test in DMEM at 37 °C (n=3). Data are expressed as mean ± SD.


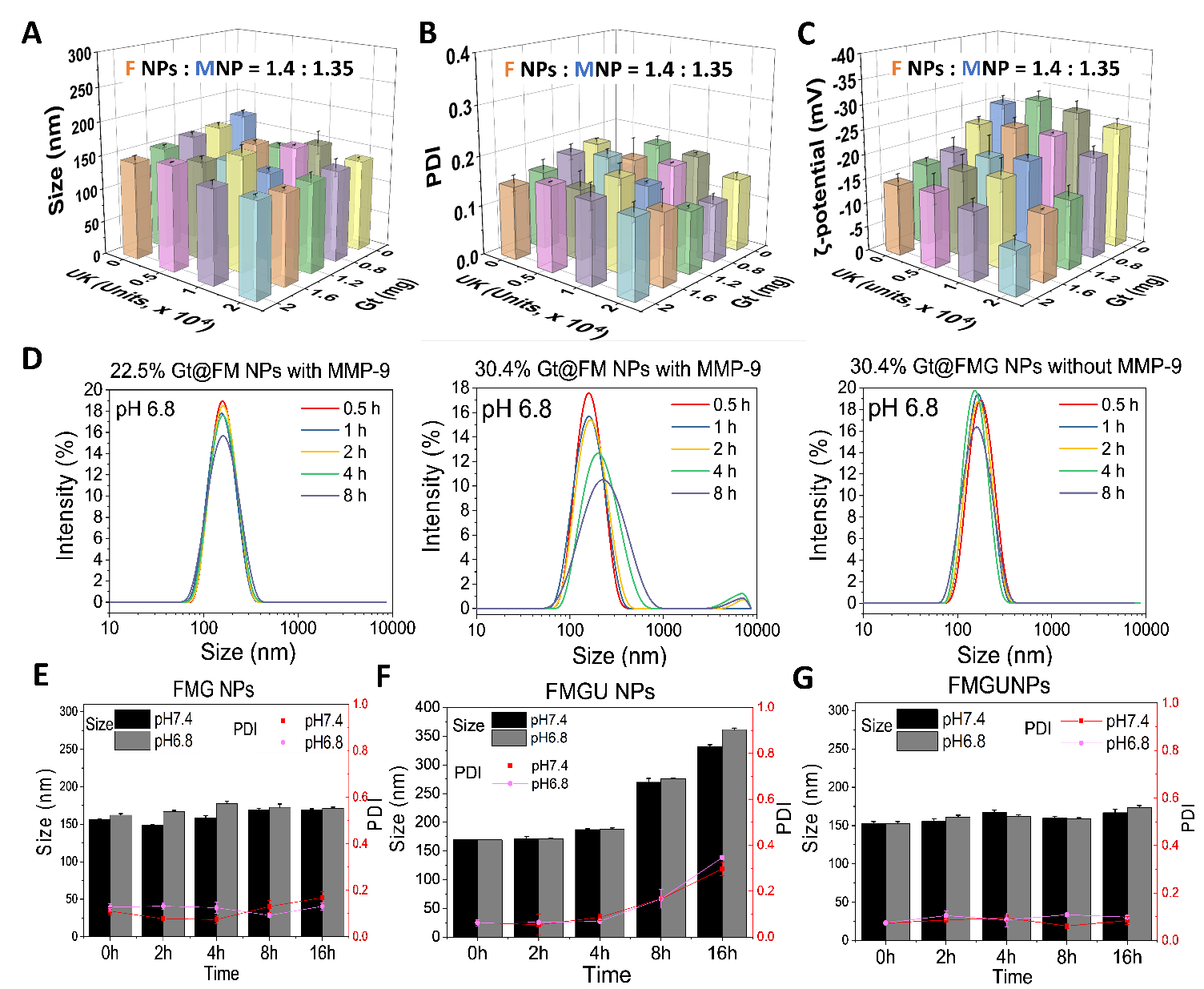


**Figure S3** Preparation and optimization of FMG NPs and FMGU NPs. (A) Mean particle size (*n*=3). (B) Polydispersity Index (PDI) (*n*=3). (C) ζ-potential (*n*=3). (D) Size distribution of different content Gt assembled with FM NPs in pH 6.8 PBS at 37°C, with or without MMP-9 (0.75 µg/mL). (E-G) Stability test in DMEM at 37 °C. (F, F NPs (mg) : MNP (mg): Gt (mg): Uk (IU) = 1.4 : 1.35 : 1.6 : 40000; G, F NPs (mg): MNP (mg): Gt (mg): Uk (IU) = 1.4 : 1.35 : 1.6 : 20000) (*n*=3). Data are expressed as mean ± SD.


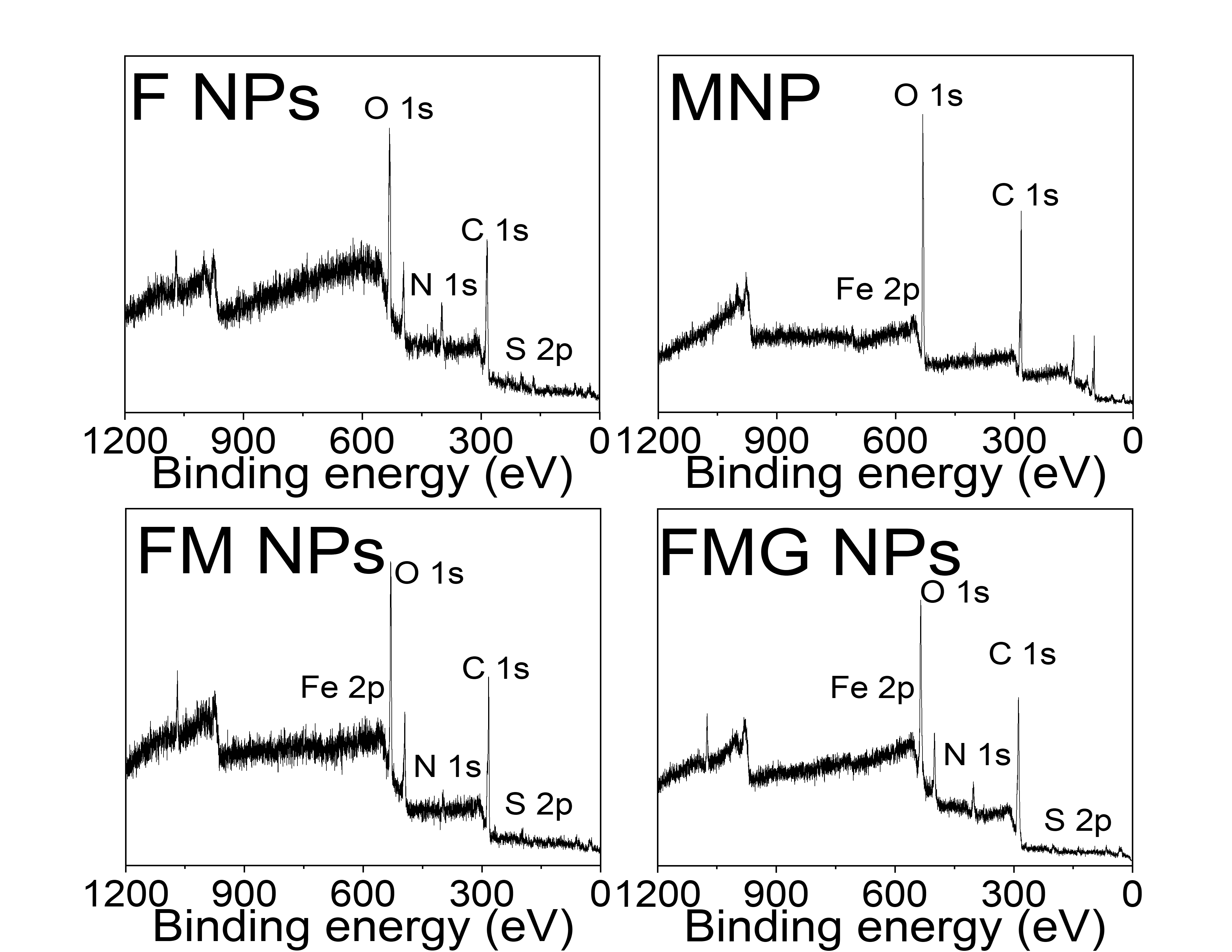


**Figure S4** XPS survey spectra of F NPs, MNP, FM NPs, and FMG NPs, showing the overall elemental composition of each formulation.

**
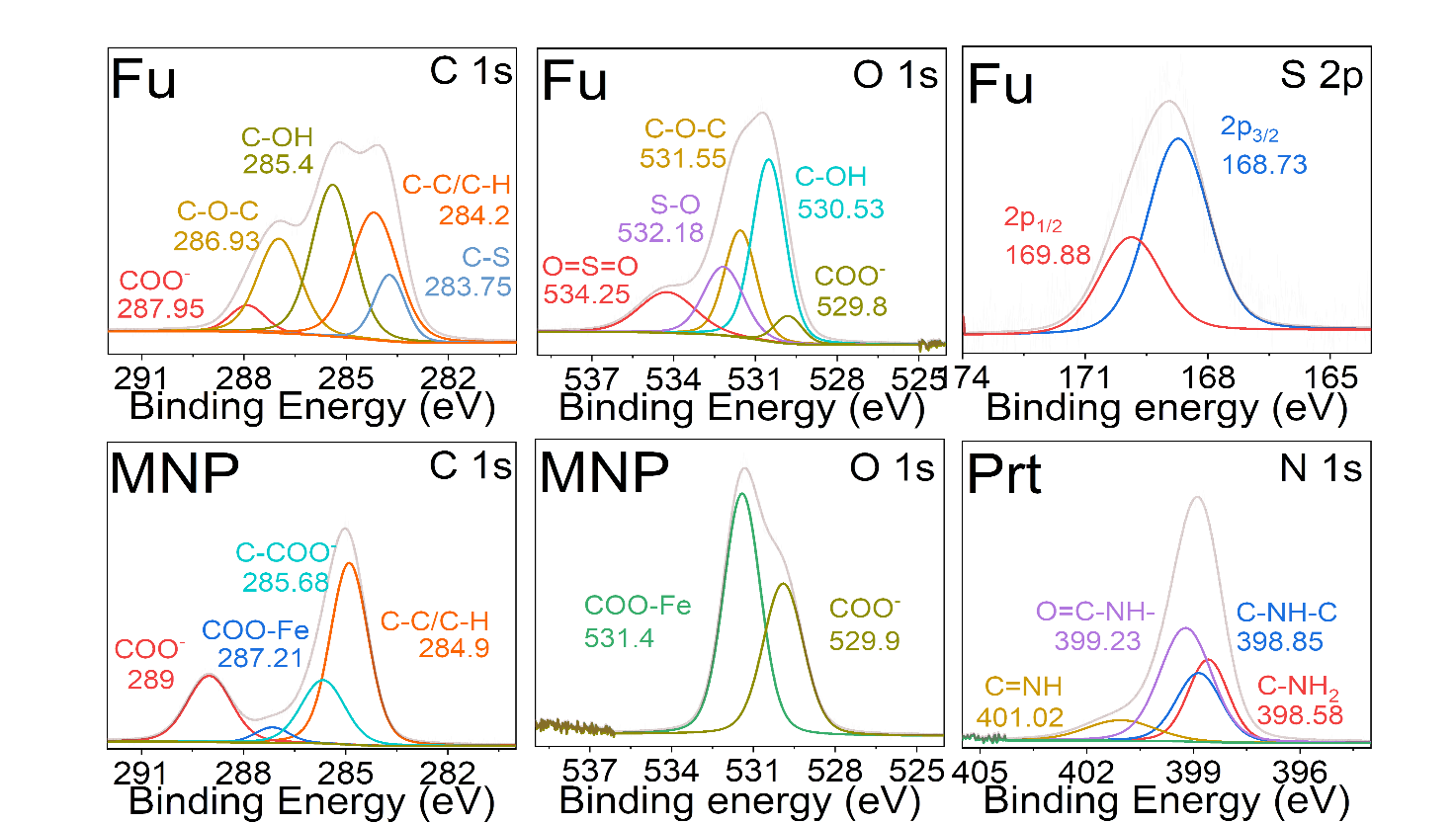
**

**Figure S5** High-resolution XPS spectra (C1s, O1s, N1s, and S2p) of Fu, MNP, and Prt.


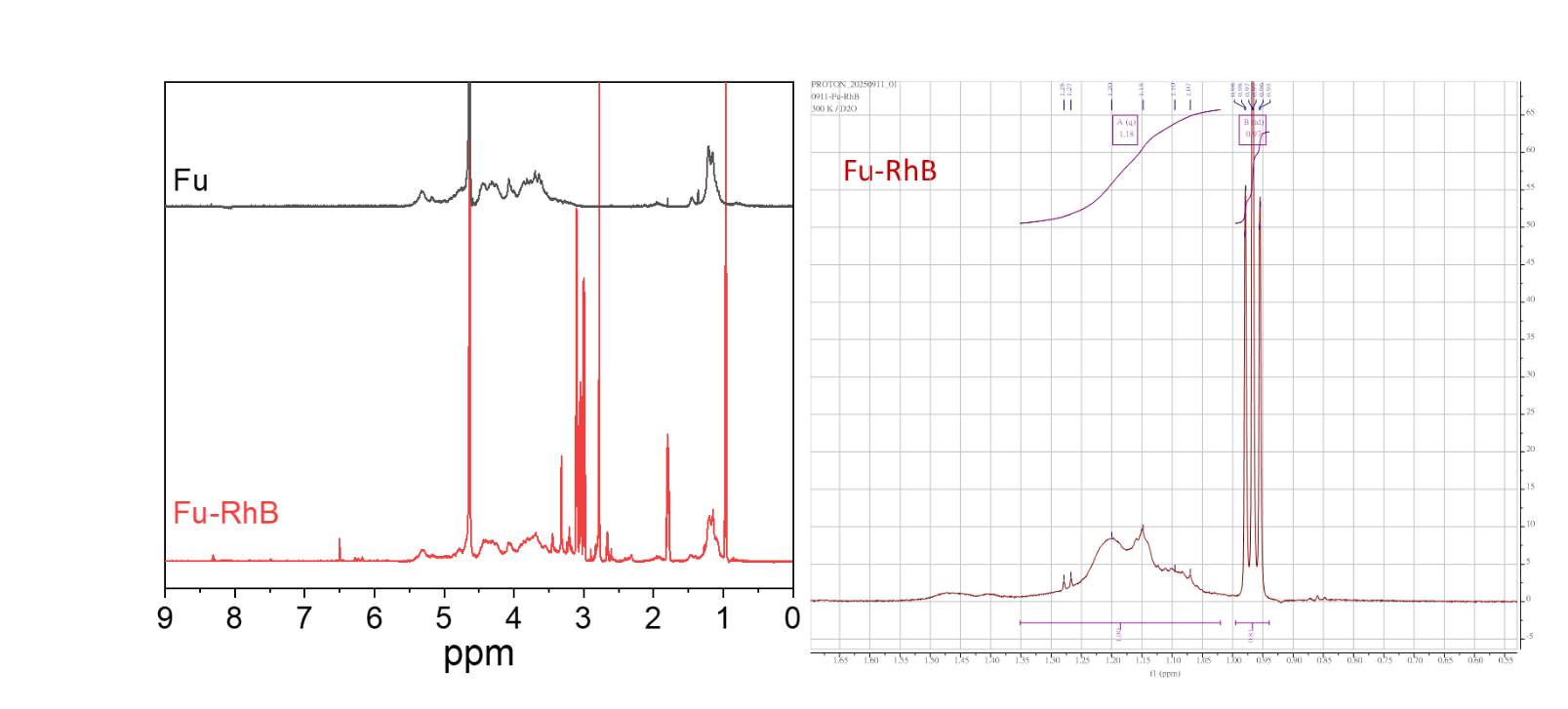


**Figure S6** Left: ^1^H NMR spectra of fucoidan (Fu, black) and Rhodamine B-conjugated Fu (Fu-RhB, red) in D_2_O. Compared to Fu, Fu-RhB exhibits new aromatic signals in the range of δ = 6.0-8.5 ppm, corresponding to the protons of Rhodamine B; a peak at δ ≈ 2.8 ppm, attributed to the methylene groups of the ethylenediamine linker; and a peak at δ ≈ 1.0 ppm, assigned to the methyl groups of Rhodamine B. In addition, chemical shift changes and peak broadening were observed in the sugar ring region (δ = 3.5-4.5 ppm), suggesting altered electronic environments near the Fu backbone upon covalent modification. These spectral features collectively support the successful conjugation of RhB onto Fu. *Right:* Zoom-in of the integration regions used for DS calculation. The degree of substitution (DS) of RhB on Fu was determined by ^1^H NMR integration. The methyl proton signal of Rhodamine B at δ ≈ 1.2 ppm (integrated to 0.81) corresponds to one of the four methyl groups (each 3H) on the RhB molecule, and was normalized by dividing by 3 × 4 = 12. The methyl signal of fucose at δ ≈ 1.3 ppm was integrated to 1.00, and divided by 3 to normalize for the 3H on the CH_3_ group of fucose. Given that fucose accounts for 22.7% of the monosaccharide units in Fu, the total sugar unit count was estimated by dividing by 0.227. The degree of substitution was then calculated as: DS% = [0.81/(3x4)] / [(1/3)/0.227] x 100% = 4.59%. This corresponds to a RhB substitution degree of approximately 4.6 %, or one RhB molecule per ~22 sugar units.

**
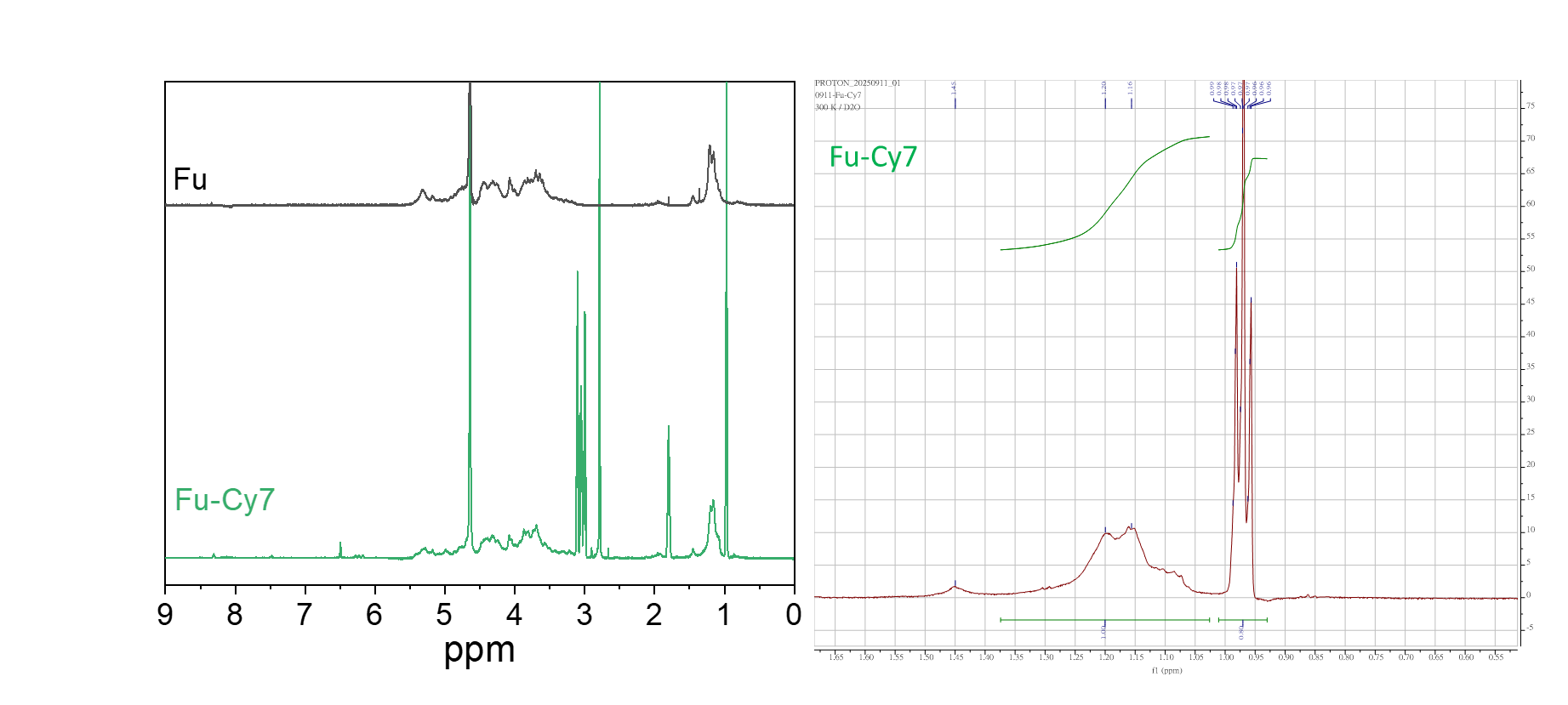
**

**Figure S7** *Left:* ^1^H NMR spectra of Fu (black) and Cy7-conjugated Fu (Fu-Cy7, green) in D_2_O. Compared to Fu, Fu-Cy7 exhibits new aromatic signals in the range of δ = 6.0-8.5 ppm, corresponding to the aromatic protons of the Cy7 core structure; a peak at δ ≈ 2.8 ppm, attributed to the methylene protons in the alkyl linker segment of the Cy7-amine; and a peak at δ ≈ 1.0 ppm, assigned to the methyl groups of the Cy7 molecule. In addition, chemical shift changes and peak broadening were observed in the sugar ring region (δ = 3.5-4.5 ppm), indicating altered electronic environments of the Fu backbone upon covalent conjugation. These spectral features support the successful attachment of Cy7 to Fu. *Right:* Zoom-in of the integration regions used for DS calculation. The methyl proton signal of Cy7 at δ ≈ 1.2 ppm (integrated to 0.80) corresponds to one of the five methyl groups (each 3H) on the Cy7 molecule. The integral was normalized by dividing by 3 × 5 = 15. The methyl signal of fucose at δ ≈ 1.3 ppm was integrated to 1.00 and divided by 3 to account for 3H per CH_3_. Given that fucose constitutes 22.7% of the monosaccharide units in Fu, the total sugar unit count was estimated by dividing by 0.227. The degree of substitution (DS) was calculated as: DS% = [0.80/(3x15)] / [(1/3)/0.227] x 100% = 3.63%. This corresponds to a Cy7 substitution degree of approximately 3.6 %, or one Cy7 molecule per ~27 sugar units.

**Table S1** Particle size, PDI, ζ-potential, and Encapsulation efficiency (EE%) of nanoparticles.

|  | **Size (nm)** | **PDI** | **ζ-potential (mV)** | **% E.E. of MNP** | **% E.E. of Uk** | **Loading content (IU Uk/mg NPs)** |
| --- | --- | --- | --- | --- | --- | --- |
| **F NPs** | 210.9 ± 1.5 | 0.23 ± 0.01 | -22.4 ± 1.1 | - | - | - |
| **FM NPs** | 170.2 ± 6.1 | 0.11 ± 0.01 | -25.6 ± 1.5 | 66.8 ± 5.4 | - | - |
| **FMG NPs** | 152.4 ± 2.2 | 0.13 ± 0.02 | -16.8 ± 0.9 | 82.3 ± 4.2 | - | - |
| **FMU NPs** | 136.4 ± 2.2 | 0.14 ± 0.01 | -26.4 ± 1.6 | 70.3 ± 2.6 | 80.3 ± 3.4 | 5836 ± 247 |
| **FMGU NPs** | 133.7 ± 2.5 | 0.13 ± 0.01 | -13.5 ± 0.9 | 85.3 ± 3.8 | 84.1 ± 2.6 | 3860 ± 119 |

^1^Each value is expressed as mean ±standard deviation (*n* = 6).

**Table S2.** Comparison of thrombus ages diagnosis capability.

| Methods | Temporal changes (the two with the largest differences were selected, fold) | Thrombus Model | Correlation between thrombus ages and signals |  |
| --- | --- | --- | --- | --- |
| black-blood MRI sequence | T1: ~1.6 (1 D - 6 W)  T2: ~2.1 (1 D - 6 W) | Porcine carotid arteries (balloon inﬂations) | non-provide | ^1^ |
| 3D-gradient echo sequence (T1)  fast asymmetric spin echo sequence (T2) | T1: ~1.1 (1 W - 4W)  T2: ~4.3 (4h - 4W) | Rabbit jugular vein (balloon catheter) | non-provide | ^2^ |
| Ultrasound elastography | ~3.1 (6D - 12D) | Porcine inferior Vena cava (ligature + thrombin) | non-provide | ^3^ |

^1^ Listed with other thrombus ages diagnosis methods reported in the literatures.

**Table S3.** Comparison of precision release strategies in urokinase-delivered carrier.

| Trigger Type | Targeting method | Free drug → particles  (remained thrombus)  Efficency enhance (fold) | Uk | Clot ages when treatment introduce / sample after treatment | Thrombus Model |  |
| --- | --- | --- | --- | --- | --- | --- |
| US + NIR | Magnetic field propelled targeting | 75.7% → 59.5%  1.3 fold | - | - | FeCl_3_ injury  (rat femoral vein) | ^4^ |
| Shear Stress | P-selectin targeting  (Fu) | 26.52% → 9.37 %  2.83 fold | - | 10 min / 2h | FeCl_3_ injury  (rat carotid artery) | ^5^ |
| NIR | Platelet targeting (tirofiban) | 38.9% → 10.4 %  3.74 fold | 32000 IU / kg | 3 min / 24h | FeCl_3_ injury  (rat femoral vein) | ^6^ |
| H_2_O_2_ | Platelet targeting (RGD) | 44.4% → 27.2 %  1.63 fold | 600 µg / kg | 5 min / 24h | FeCl_3_ injury  (rat femoral vein) | ^7^ |
| MMP-2/9 | P-selectin targeting  (Fu) | 34.1% → 3.5%  9.66 fold | 64102 IU / kg | 180 min / 3h | FeCl_3_ injury  (mice carotid artery) | This study |

^1^ Listed with other urokinase-load carriers for thrombolysis treatment reported in the literatures.

^2^ US: Ultrasound; NIR: Near‐Infrared

**Experimental Section**

*Materials*: Fucoidan (Fu; derived from *Laminaria japonica*, with a molecular weight of 40 kDa, containing 35% fucose, 28% sulfate ester groups, and 10% carboxyl groups) was obtained from NOVA Pharma & Biotech (Kaohsiung, Taiwan). Protamine (Prt; derived from salmon), type A gelatin (Gt; from porcine skin), ferric chloride, and poly(acrylic acid) (PAA) were purchased from Sigma-Aldrich (St. Louis, MO, USA). Urokinase (Uk) was kindly provided by China Chemical & Pharmaceutical (Taipei, Taiwan).

*Synthesis of MNPs*: PAA-coated magnetite nanoparticles (PAA-Fe_3_O_4_ NPs) were synthesized via a chemical coprecipitation method. A 0.4% (w/v) PAA solution was prepared by dissolving 0.32 g of PAA (Mn 1800 Da, Sigma-Aldrich) in 80 mL of deionized water and preheating the solution to 80 °C under magnetic stirring (700 rpm). Separately, FeCl_3_·6H_2_O (0.1825 g, 0.54 mmol) and FeCl_2_·4H_2_O (0.06925 g, 0.279 mmol) were dissolved in 2.5 mL of 1 M HCl to form the iron precursor solution. This precursor was rapidly injected into the preheated PAA solution under vigorous stirring. Subsequently, 5 mL of concentrated ammonia solution (NH_4_OH, ~28%) was added dropwise to adjust the pH to 9-10. The reaction mixture was stirred at 80 °C for 1 h, then cooled to room temperature. The resulting black suspension was purified by dialysis (MWCO 10,000 Da) against deionized water for 48 h, with water changes every 6-8 h. The purified nanoparticles were lyophilized and weighed to determine the dry yield, which was approximately 82.3 ± 5.7% (*n* = 3), calculated as the weight of lyophilized product divided by the total theoretical mass of iron salts used in the reaction. The product was designated as PAA-Fe_3_O_4_ NPs (MNPs). Magnetic measurements were performed using a superconducting quantum interference device (SQUID, Quantum Design, Germany) at 300 K under magnetic fields ranging 0-200 Oe (at intervals of 20 Oe), 400-2000 Oe (at intervals of 200 Oe), and 3000-20,000 Oe (at intervals of 1000 Oe).

*Preparation and characterization of NPs:* Fu, Prt, and Gt were prepared as solutions in phosphate-buffered saline (PBS) at a concentration of 4 mg/mL, with the pH adjusted to 7.4. Uk was prepared as a solution at 200,000 IU/mL, also adjusted to pH 7.4. MNPs were suspended in PBS at a concentration of 45 mg/mL. Based on electrostatic interactions, positively charged components (Prt, Gt, and Uk) were first pre-mixed, and negatively charged components (Fu and MNPs) were also pre-mixed separately. These two mixtures were then combined under gentle vortexing at room temperature and incubated for 3 min to allow complete self-assembly. The formulations were screened and optimized by adjusting volumetric ratios. The assembled nanoparticle suspension was then transferred to 300 kDa molecular weight cutoff ultrafiltration tubes (Vivaspin 2^®^, Sartorius, USA) and centrifuged at 3000 rpm to remove unassembled components and free drug. The retained nanoparticles were washed three times using the same ultrafiltration protocol with PBS, and collected for subsequent analysis.

The average particle size, size distribution, ζ-potential, and polydispersity index (PDI) were measured using a ZEN3600 dynamic light scattering system (DLS, Malvern, Worcestershire, UK). All DLS measurements were performed in PBS (pH 7.4) at 25 °C or 37 °C without dilution, with each sample measured 12 times using disposable cuvettes. For TEM analysis, nanoparticle suspensions were dropped onto carbon-coated copper grids, vacuum-dried, and imaged at an accelerating voltage of 100 kV, using an H-7700 transmission electron microscope (Hitachi, Tokyo, Japan). For the magnetic resonance imaging (MRI) analysis, a 0.2-mL PCR tubes encased in modeling clay were filled completely with nanoparticle suspension to minimize air interfaces during imaging. MRI parameters were set to retention time (TR) of 100 ms and echo time (TE) of 3 ms (FISP) for T1-weighted imaging, and TR of 3000 ms and TE of 48 ms (spin-echo high-resolution (highres)) for T2-weighted imaging, performed using a Bruker Pharmascan 7T system (Bruker, Ettlingen, Germany). Chemical characterization was conducted using FTS-155 Fourier-transform infrared (FTIR) spectroscopy (Bio-Rad, Hercules, CA, USA) and high-resolution X-ray photoelectron spectrometry (XPS, ULVAC-PHI, PHI Quantera II, Tokyo, Japan). FTIR was performed using lyophilized samples prepared as KBr pellets, and XPS analysis was conducted on suspensions vacuum-dried onto clean silicon wafers.

*Stability, and drug-loading efficiency and release*: For the stability analysis, nanoparticles were suspended in PBS at pH 7.4 or pH 6.8 at a sample-to-buffer ratio of 2:8 and incubated at 37 °C. At specific time points, samples were centrifuged at 14,000 rpm for 15 min. The average particle size and PDI were measured at each time point using DLS. For the drug-release analysis, the nanoparticles were suspended in PBS at pH 7.4 or pH 6.8 at a sample-to-medium ratio of 2:8 and incubated at 37 °C. At predetermined intervals, samples were centrifuged at 14,000 rpm for 15 min, and the supernatant was collected for analysis. The same volume of fresh PBS was added to replace the sampled medium at each time point. MNP release was quantified by measuring the absorbance at 490 nm. Uk release was quantified using a Dionex Ultimate 3000 UHPLC (ultrahigh performance liquid chromatographic) system equipped with an Ascentis Express C18 column (4.6 × 150 mm, 90 Å, 2.7 µm; Supelco, Bellefonte, PA, USA) and an ultraviolet-visible (UV-Vis) detector (Thermo Scientific, MA, USA). Chromatographic separation was performed at 225 nm with a flow rate of 1 mL/min using gradient elution. The mobile phase consisted of (A) 0.1% trifluoroacetic acid in deionized water and (B) 0.1% trifluoroacetic acid in methanol. The gradient program was as follows: 0–3 min, 0%–40% (B); 3–15 min, 40%–70% (B); 15–24 min, 70%–80% (B); and 24–25 min, 80%–100% (B). Encapsulation efficiency (EE) was calculated using equation (1). The amount of drug released over time was determined using equations (2) and (3).

Encapsulation efficiency (EE%) was calculated as:

$$(1) EE (\%)=\left( \frac{W_{\mathrm{total}}-W_{\mathrm{free}}}{W_{\mathrm{total}}} \right)\times100$$

*Where*, *W*_total_: total amount of drug initially added to the formulation. *W_free_*​: amount of unencapsulated (free) drug measured in the supernatant

Cumulative drug release (%) was calculated by:

$$(2) Pt=\left( \frac{C_{t}\times V}{W_{\mathrm{total}}} \right)\times100$$

$$(3) Cumulative release (\%)=P_{t}+P_{(t - 1)}$$

*Where, C_t​_*: drug concentration in the supernatant at time point t. *V*: volume of release medium. *W_total​_*: total amount of drug encapsulated in nanoparticles. *P_t_*​: percentage of drug released at time point t. *P_t−1_​*: cumulative percentage of drug released up to the previous time point *t−1*. Since the release medium was replaced with fresh PBS after each time point to maintain sink conditions, cumulative release was calculated by summing the newly released fraction (*P_t_*​) with the cumulative release from the previous time point (*P_t−1_​*).

*Thrombus-binding capability and in vitro MRI*: Fu-RhB was synthesized through a two-step EDC/NHS-mediated coupling process. First, 1.0 g of Fu was dissolved in 200 mL of deionized water (0.5% w/v), and the pH was adjusted to 5.5. To activate the carboxyl groups on Fu, 0.5 g of EDC and 0.25 g of NHS were added and stirred for 2 h at room temperature. After activation, 40 μL of ethylenediamine was added, and the reaction proceeded for 24 h at room temperature. The mixture was dialyzed against deionized water (MWCO 10 kDa) for 2-3 days to remove unreacted ethylenediamine and by-products. The resulting Fu-ethylenediamine intermediate (Fu-NH_2_) was lyophilized and stored at −20 °C. In the second step, a 0.01% (w/v) solution of rhodamine B isothiocyanate (Chem-Impex, Wood Dale, USA) in DMSO was prepared and added dropwise to the Fu-NH_2_ solution. The reaction was carried out at room temperature for 24 h under light-protected conditions. After the reaction, the mixture was dialyzed against deionized water (MWCO 10 kDa) for 2-3 days and lyophilized to obtain Fu-RhB. The labeling efficiency of RhB on Fu was determined to be approximately 0.4%. Successful conjugation was confirmed by ^1^H NMR analysis (Fig. S6). Fu-RhB was subsequently used in nanoparticle assembly.

Thrombus preparation followed ARRIVE guidelines and was approved by the Taipei Medical University Animal Ethics Committee (LAC-2022-0150). Fresh blood was collected from the tail artery of SD rats and preserved in 10% citrate-phosphate-dextrose (CPD) buffer (16 mM citric acid, 90 mM sodium citrate, 16 mM NaH_2_PO_4_, and 142 mM glucose; pH 7.4) before use. Platelet-rich plasma (PRP) was prepared by centrifugation of preserved blood. Clot formation was initiated by mixing whole blood or PRP with 2.5 mM calcium chloride and 1 U/mL thrombin, followed by incubation at 37 °C for at least 1 h to promote thrombus formation. For the binding analysis, RhB-labeled nanoparticles were incubated with the prepared thrombus samples at 37°C. Samples were blocked with a 3% bovine serum albumin solution, followed by treatment with an anti-P-selectin antibody (CD62P, 1:50 dilution; sc-8419, Santa Cruz Biotechnology, CA, USA) or 1% Fu for 2 h. Subsequently, the thrombus samples were treated with nanoparticles for 2 h, washed with PBS, and analyzed using the PerkinElmer IVIS Lumina XRMS Series III Imaging System (Waltham, MA, USA). Quantification was performed using PerkinElmer IVIS Lumina XRMS imaging software. For the MRI analysis, samples prepared in tubes were overlaid with 2% agarose. The tubes were wrapped with clay for stabilization and arranged in parallel for imaging. MRI was performed to assess thrombus-binding capabilities.

*In vivo aging thrombus model*: All animal procedures were conducted in compliance with ARRIVE guidelines and approved by the Taipei Medical University Animal Ethics Committee (LAC-2022-0150). The aging thrombus model was established using 8-week-old ICR mice (Lasco, Taipei, Taiwen), which were randomly assigned to groups. Under anesthesia with 1% isoflurane gas, the carotid artery was surgically exposed and supported with a paper strip. Filter paper (1 × 2 mm) soaked in a 10% ferric chloride solution was applied to one side of the exposed carotid artery for 3 min. Following removal of the filter paper, the area was thoroughly rinsed with PBS. The incision was sutured, and mice were housed under standard conditions until the designated time for tissue collection and sacrifice. Both carotid arteries were excised and embedded in paraffin to prepare 4-μm thick sections for the immunohistochemical (IHC) analysis. IHC staining was performed using the BOND-MAX fully automated IHC staining systems (Leica Microsystems, Wetzlar, Germany). Briefly, sections were deparaffinized and rehydrated, and then incubated with 3% hydrogen peroxide for 5 min to block endogenous peroxidase activity. After washing, antigen retrieval was performed in an EDTA solution for 40 min. Tissue sections were incubated with primary antibodies against MMP-2 (1:100 dilution; Abclonal, A6247), MMP-9 (1:100 dilution; Abclonal, A0289), CD62P (1:500 dilution; Cohesion, CPA5429), and CD204 (1:500 dilution; Cusabio, CSB-PA194131). Antigen retrieval was performed using Tris-EDTA buffer (pH 9.0, 12 min) for MMP-2/MMP-9 and citrate buffer (pH 6.0, 12 min) for CD62P, while no retrieval was required for CD204. After blocking for 60 min, sections were incubated with primary antibodies at room temperature (MMP-9 for 2 h; MMP-2, overnight; CD62P and CD204, 1 h). After washing, sections were incubated with fluorophore-conjugated secondary antibodies (anti-rabbit Alexa Fluor 488 or 594; TATS01F kit, Taiwan Advanced Technology, Taipei, Taiwan) for 30 min at room temperature. Nuclei were counterstained with DAPI for 1 min, and sections were mounted for microscopic examination.

Total RNA was extracted from thrombotic and contralateral uninjured carotid arteries using TRI Reagent^®^ (Sigma-Aldrich, USA) according to the manufacturer’s instructions. The concentration and purity of RNA were assessed spectrophotometrically (Nanophotometer N60, Implen, Munich, Germany). Subsequently, 500 ng of total RNA was reverse-transcribed into cDNA using the ExcelRT^TM^ Reverse Transcription Kit (SMOBIO, Taiwan). Quantitative PCR was performed using ExcelTag^TM^ 2X Fast Q-PCR Master Mix (SYBR Green, no ROX; SMOBIO) on a LightCycler^®^ 480 System (Roche, Switzerland). Commercially validated primer sets targeting mouse *Mmp2*, *Mmp9*, and *Actb* were synthesized by Sigma-Aldrich. All reactions were carried out in triplicate (*n* = 3). The threshold cycle (Ct) values of the target genes were normalized to the housekeeping gene *Actb*, and relative gene expression levels were calculated using the 2^–ΔΔCt^ method. Gene expression in thrombotic samples was expressed as fold change relative to the contralateral healthy artery (defined as 1.0). The primer sequences used were as follows: *Mmp2*, (F) 5'-GAGATCTTCTTCTTCAAGGAC-3', (R) 5'-AATAGACCCAGTACTCATTCC-3'; *Mmp9*, (F) 5'-CTTCCAGTACCAAGACAAAG-3', (R) 5'-ACCTTGTTCACCTCATTTTG-3'; *Actb*, (F) 5'-GATGTATGAAGGCTTTGGTC-3', (R) 5'-TGTGCACTTTTATTGGTCTC-3'.

*Targeting and biodistribution*: Fu-Cy7 was synthesized by EDC/NHS-mediated conjugation. Briefly, 0.5 g of Fu was dissolved in 100 mL of deionized water containing 0.9 g MES buffer and 5 mL DMSO. The pH was adjusted to 5.5. To activate the carboxyl groups on Fu, 250 mg of EDC and 125 mg of NHS were added and stirred for 2 h at room temperature. Then, 6.25 mg of Cy7-amine (in DMSO; Lumiprobe, Hannover, PA, USA) was added dropwise under light-protected conditions, and the reaction proceeded at room temperature for 2 days in the dark. After the reaction, the mixture was dialyzed using a membrane (MWCO 10 kDa) against deionized water for 3 days, with frequent water changes. The resulting solution was lyophilized to obtain Fu-Cy7. Successful conjugation was confirmed by ¹H NMR analysis (Fig. S7). Fu-Cy7 was subsequently used in nanoparticle assembly.

Cy7-labeled nanoparticles were administered via a tail vein injection. Real-time observations were performed at specified time points using the PerkinElmer IVIS (*in vivo* imaging system) Lumina XRMS Series III Imaging System. To reduce background interference during imaging, a piece of black paper was passed through both carotid arteries to provide clearer visualization. Throughout the experiment, mice were maintained on a 37 °C warming plate under anesthesia with 1% isoflurane. Ninety minutes post-injection, the carotid arteries and major organs (heart, liver, spleen, lungs, and kidneys) were harvested and imaged using the IVIS system equipped with a Cy7 filter for qualitative and quantitative analyses. For the co-localization analysis, tissues were embedded in Tissue-Tek^®^ O.C.T. compound (Sakura Finetek, Torrance, CA, USA) 1 h after the nanoparticles injection. Embedded tissues were frozen, sectioned using a Leica CM3050S cryostat (Leica, Wetzlar, Germany), and stained with DAPI, CD62P (1:500 dilution, Cohesion), or CD204 (1:500 dilution, Cusabio). Stained sections were then mounted and visualized using a Leica Stellaris 8 confocal imaging system (Leica Microsystems).

*In vivo MRI:* After inducing carotid artery thrombosis, an ear bar and retention needle were used to stabilize the imaging position. Mice were anesthetized with 1% isoflurane, and their heart rate and respiration were continually monitored. Animals were then positioned in a Bruker Pharmascan 7T system (Bruker, Ettlingen, Germany) for T1-weighted and T2-weighted MRI. Initial MRI scans of the carotid artery thrombosis were performed prior to the nanoparticles injection. The nanoparticles were then administered via the retention needle, and imaging scans commenced 2 h post-injection. For T1-weighted imaging, the slice thickness was set to 0.75 mm, the TR to 200 ms, and the TE to 3.1 ms. For T2-weighted imaging, the slice thickness was 0.75 mm, with a TR of 2500 ms and a TE of 33 ms.

*Thrombolysis:* Blood was collected from the tail artery of rats. A pro-coagulant solution was prepared by mixing 5 μL of 480 mM calcium chloride (final concentration: 20 mM) with 15 μL of 120 U/mL thrombin (final concentration: 18 U/mL). To form thrombi *in vitro*, 5 μL of the pro-coagulant solution was placed at the bottom of each well in a 96-well plate, forming a ring. Next, 25 μL of rat whole blood was mixed with the pro-coagulant solution along the bottom edge of each well. The plate was incubated at 37 °C for 4 h to ensure complete thrombus formation. For the *in vivo* thrombolysis analysis, the previously established carotid artery thrombosis model was used. Nanoparticles were injected via a tail vein. Three hours post-injection, a paper strip was inserted under the carotid artery to expose it for imaging. Laser speckled contrast imaging (RWD, Sugar Land, TX, USA) was employed to record blood flow images and analyze the blood flow intensity. To evaluate the thrombus size, carotid arteries were collected and stained with hematoxylin and eosin (H&E). The thrombus size was assessed and quantified using ImageJ software (National Institutes of Health, Bethesda, MD, USA).

*Hemorrhaging, hemolysis, and biosafety:* For the rodent tail-bleeding assay, all animal procedures were conducted in compliance with ARRIVE guidelines and approved by the Taipei Medical University Animal Ethics Committee (LAC-2024-0080). Carotid artery thromboses were induced to form in SD rats using the FeCl_3_ method, followed by a tail vein injection of Uk or FNG NPs (64,102 IU Uk/kg body weight (BW)). After 1 h, the distal 10 mm of the rat tail was excised, and blood was collected on filter paper or the tail was immersed in 37 °C PBS for 30 min to measure hemoglobin absorbance.

For the hemolysis assay, Nanoparticles were mixed with freshly collected blood containing 3.2% sodium citrate as an anticoagulant and incubated at 37 °C for 1 h. After incubation, samples were centrifuged at 1000 *g* for 10 min, and the supernatant was collected. Hemoglobin released into the supernatant was quantified by measuring the absorbance at 540 nm.

For the biosafety evaluation (LAC-2022-0150), Nanoparticles were administered via a tail vein injection at a dose equivalent to 64,102 IU Uk/kg BW in ICR mice, once weekly for four consecutive weeks. Major organs (heart, liver, spleen, lungs, and kidneys) were collected and assessed for toxicity using H&E staining. Stained tissues were examined for histopathological changes to identify any potential toxicity or adverse effects. Alanine aminotransferase (ALT), aspartate aminotransferase (AST), alkaline phosphatase (ALP), lactate dehydrogenase (LDH), creatinine (CREA), and blood urea nitrogen (BUN) levels were measured using the IDEXX Catalyst^®^ One Analyzer (IDEXX Laboratories, Westbrook, Maine, USA).

To evaluate the *in vivo* clearance of MNPs and Fu, elemental analysis of Fe and S was performed in major organs and thrombosed vessels collected at 4 weeks post-injection. Tissues were lyophilized, weighed, and digested in concentrated nitric acid (HNO_3_) using a high-pressure digestion vessel at 180 °C for 60 min, as previously described ^8^. The resulting digests were diluted with ultrapure water and analyzed using an inductively coupled plasma mass spectrometer (ICP-MS/MS, Agilent 8900, Agilent, Waldbronn, Germany) coupled with an Agilent 1260 Infinity Quaternary LC System. Fe and S were quantified as representative elemental markers for MNPs and Fu, respectively.

The cytocompatibility of the nanoparticles was assessed using human umbilical vein endothelial cells (HUVECs). HUVECs were cultured in EBM-2 medium supplemented with the Endothelial Growth Medium (EGM)-2 SingleQuots kit (Lonza, Tokyo, Japan). Cell viability was evaluated using an MTT assay, with absorbance measured at 570 nm on a PerkinElmer plate reader (EnSpire 2300).

*Statistical analysis*: All experimental data are expressed as the mean ± standard deviation (*n* = 3–8). Statistical analysis was performed using Student's *t*-test. *p* values of < 0.05 (* *p* < 0.05), < 0.01 (** *p* < 0.01), and < 0.001 (*** *p* < 0.001) were considered statistically significant. All statistical analyses were conducted using SPSS vers. 22.0 (IBM, Armonk, NY, USA).

**Reference**

1. Corti R, Osende JI, Fayad ZA, et al. In vivo noninvasive detection and age definition of arterial thrombus by MRI. J Am Coll Cardiol. (2002). <https://doi.org/10.1016/s0735-1097(02)01754-0>

2. Kuroiwa Y, Yamashita A, Miyati T, et al. MR signal change in venous thrombus relates organizing process and thrombolytic response in rabbit. Magn Reson Imaging. (2011). <https://doi.org/10.1016/j.mri.2011.04.015>

3. Geier B, Barbera L, Muth-Werthmann D, et al. Ultrasound elastography for the age determination of venous thrombi. Thromb Haemost. (2005). <https://doi.org/10.12659/PJR.899517>

4. Ruan R, Chen S, Su J, et al. Targeting Nanomotor with Near‐Infrared/Ultrasound Triggered‐Transformation for Polystage‐Propelled Cascade Thrombolysis and Multimodal Imaging Diagnosis. Adv Healthc Mater. (2024). <https://doi.org/10.1002/adhm.202302591>

5. Zhang H, Pei Y, Gao L, et al. Shear force responsive and fixed-point separated system for targeted treatment of arterial thrombus. Nano Today. (2021). <https://doi.org/10.1016/j.nantod.2021.101186>

6. Liu S, Sun Y, Zhang T, et al. Upconversion nanoparticles regulated drug & gas dual-effective nanoplatform for the targeting cooperated therapy of thrombus and anticoagulation. Bioact Mater. (2022). <https://doi.org/10.1016/j.bioactmat.2022.03.013>

7. Cao W, Wei W, Qiu B, et al. Ultrasound-powered hydrogen peroxide-responsive Janus micromotors for targeted thrombolysis and recurrence inhibition. Chem Eng J. (2024). <https://doi.org/10.1016/s0735-1097(02)01754-0>.

8. Tsai H-T, Lin C, Chung C-H, et al. Fucoidan-decorated metal-zoledronic acid nanocomplexes suppress tumor metastasis by inducing ferroptotic cell death and enhancing cancer immunotherapy. J nanobiotechnol. (2025). <https://doi.org/10.1186/s12951-025-03473-0>
